# Catalysts of Healing: A Symphony of Synthesis and Clinical Artistry in Small-Molecule Agents for Breast Cancer Alleviation

**DOI:** 10.3390/molecules29051166

**Published:** 2024-03-05

**Authors:** Jing Hu, Bi-Yue Zhu, Zhen-Xi Niu

**Affiliations:** 1 Children’s Hospital Affiliated to Zhengzhou University, Henan Children’s Hospital, Zhengzhou Children’s Hospital, Zhengzhou 450018, China; hujingzzu@outlook.com; 2 Martinos Center for Biomedical Imaging, Massachusetts General Hospital and Harvard Medical School, Boston, MA 02129, USA; 3 Department of Pharmacy, Children’s Hospital of Chongqing Medical University, Chongqing 400015, China

**Keywords:** breast cancer, small-molecule drugs, synthetic pathways, clinical applications

## Abstract

Breast cancer, characterized by its molecular intricacy, has witnessed a surge in targeted therapeutics owing to the rise of small-molecule drugs. These entities, derived from cutting-edge synthetic routes, often encompassing multistage reactions and chiral synthesis, target a spectrum of oncogenic pathways. Their mechanisms of action range from modulating hormone receptor signaling and inhibiting kinase activity, to impeding DNA damage repair mechanisms. Clinical applications of these drugs have resulted in enhanced patient survival rates, reduction in disease recurrence, and improved overall therapeutic indices. Notably, certain molecules have showcased efficacy in drug-resistant breast cancer phenotypes, highlighting their potential in addressing treatment challenges. The evolution and approval of small-molecule drugs have ushered in a new era for breast cancer therapeutics. Their tailored synthetic pathways and defined mechanisms of action have augmented the precision and efficacy of treatment regimens, paving the way for improved patient outcomes in the face of this pervasive malignancy. The present review embarks on a detailed exploration of small-molecule drugs that have secured regulatory approval for breast cancer treatment, emphasizing their clinical applications, synthetic pathways, and distinct mechanisms of action.

## 1. Introduction

Breast carcinoma stands as the prevailing malignancy in the female population, thereby establishing it as a subject of paramount significance within the domain of oncological research. With over 2.3 million cases diagnosed in 2020, the disease remains a significant public health challenge, illustrating the dire need for the continuous evolution of therapeutic modalities [1]. The intricacies of breast cancer, due to its molecular and genetic heterogeneity, demand tailored therapeutic strategies, emphasizing precision medicine [2]. Breast cancer treatment often involves a combination of surgery, radiation, and systemic therapies, including chemotherapy, hormonal therapy, targeted therapy, and immunotherapy. The choice of monotherapy or combination therapy depends on the specific characteristics of the tumor, its stage, and the patient’s overall health [3,4,5,6].

The past decade has witnessed a paradigm shift towards more targeted treatments, especially with the advent of small-molecule drugs, given their capacity to specifically modulate or inhibit distinct oncogenic pathways [7]. The surge in the design and synthesis of small-molecule drugs against breast cancer is primarily a culmination of advancements in drug discovery methodologies [8]. Techniques such as high-throughput screening, molecular docking, and quantum chemical calculations have facilitated the identification of potent molecules with high specificity [9]. Additionally, a deeper understanding of structural biology has empowered the rational design of these drugs to intricately engage with their target sites, often kinases or other critical protein–protein interfaces [10]. In turn, this specificity has not only presented an avenue for improved therapeutic outcomes but has also opened up potential avenues for reduced drug-associated adverse effects and mitigation of therapeutic resistance [11]. Currently, several small-molecule drugs addressing diverse pathways from hormone signaling to DNA repair mechanisms have been approved for breast cancer treatment, further expanding the therapeutic landscape. Triple-negative breast cancer (TNBC), characterized by the absence of estrogen receptor (ER), progesterone receptor (PR), and human epidermal growth factor receptor 2 (HER2), presents a formidable challenge in terms of targeted therapy due to its lack of specific molecular targets. As a result, chemotherapy remains a primary treatment approach for TNBC. Anthracyclines such as doxorubicin and taxanes like paclitaxel are commonly employed, showcasing efficacy in managing TNBC by disrupting cell division and inducing apoptosis [12]. HER2-overexpression subtype breast cancers, characterized by amplified HER2/neu gene expression, have witnessed significant therapeutic breakthroughs. Small molecule TKIs such as lapatinib, neratinib, and tucatinib are used in HER2-overexpression breast cancer treatment [13]. Hormone receptor-positive (HR+) breast cancers, expressing estrogen and/or progesterone receptors, are effectively managed with endocrine therapies. Selective estrogen receptor modulators like tamoxifen and aromatase inhibitors such as anastrozole, letrozole, and exemestane, as well as selective estrogen receptor degraders such as fulvestrant play crucial roles in inhibiting hormone-driven tumor growth [14]. The introduction of CDK4/6 inhibitors (e.g., palbociclib, ribociclib) in combination with endocrine therapy has demonstrated substantial benefits, delaying disease progression and improving overall survival in HR+ metastatic breast cancer [15].

**Figure 1:**
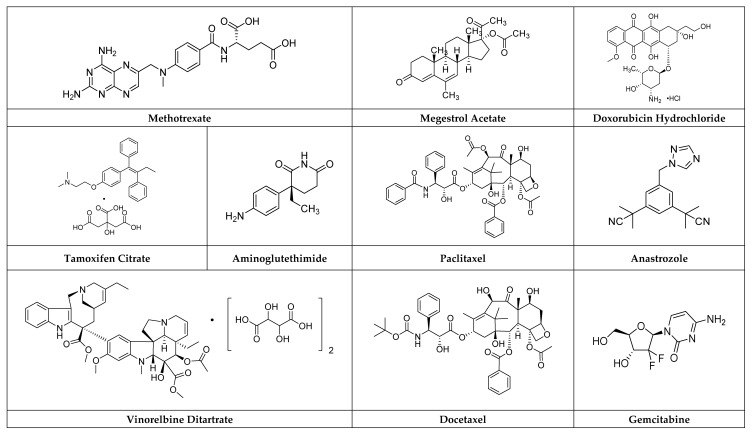

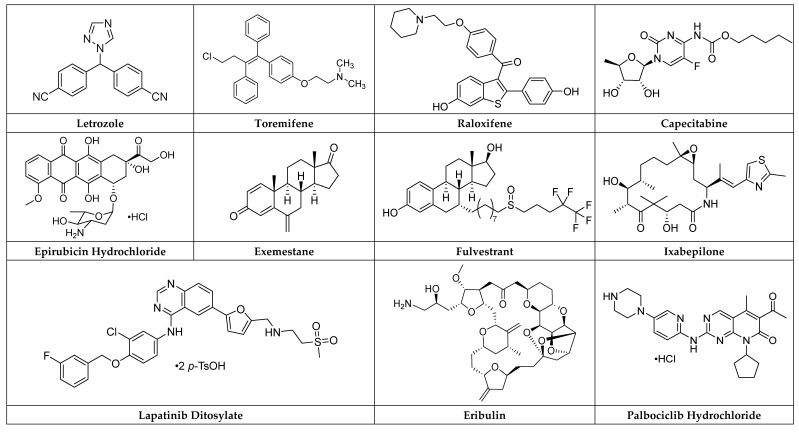

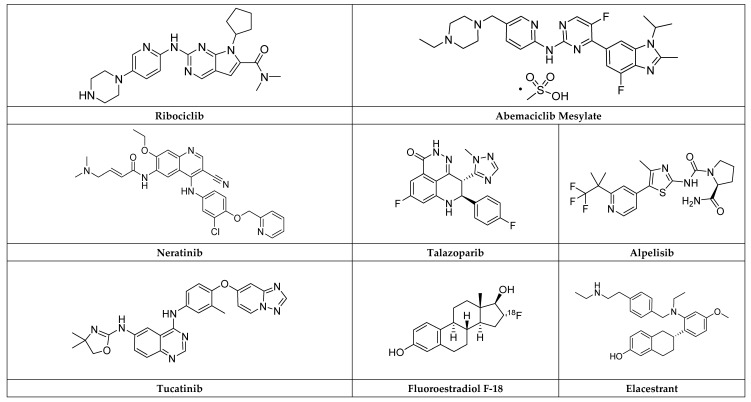
Chemical structures of representative small-molecule drugs for the treatment of breast cancer.

However, as with any therapeutic modality, small-molecule drug development and implementation come with their sets of challenges. From their pharmacokinetics and pharmacodynamics to potential drug–drug interactions, the path to regulatory approval and clinical application is intricate [16]. Additionally, while these drugs present a promising therapeutic avenue, the emergence of resistance remains a concern, underlining the need for continuous research and adaptation of these molecules [17]. This review aims to elucidate the gamut of small-molecule drugs that have garnered regulatory approval for breast cancer treatment (Table 1 and Figure 1). By meticulously examining their clinical applications, synthetic pathways, and mechanistic underpinnings, we aspire to offer a consolidated and up-to-date resource for clinicians, researchers, and drug developers.

## 2. Small-Molecule Drugs to Treat Breast Cancer

### 2.1. DNA Inhibitors

#### 2.1.1. Methotrexate

Methotrexate received regulatory approval from the U.S. Food and Drug Administration (FDA) in 1953, and was subsequently marketed under the trade designation Emtexate. Methotrexate, as a derivative of folate, exerts inhibitory effects on a multitude of enzymes pivotal in the biosynthesis of nucleotides. These enzymatic constraints result in the concurrent attenuation of inflammatory processes and the impediment of cellular proliferation [18]. In light of these pharmacological attributes, Methotrexate finds frequent application in the management of inflammatory conditions such as arthritis and in the regulation of cellular mitosis within neoplastic disorders like breast cancer and non-Hodgkin’s lymphoma. Methotrexate acts primarily as an antagonist of folic acid. It inhibits the enzyme dihydrofolate reductase, leading to a reduction in the synthesis of tetrahydrofolate. Since tetrahydrofolate is essential for the synthesis of purine and pyrimidine bases (components of DNA and RNA), Methotrexate essentially disrupts DNA synthesis, repair, and cellular replication [19,20,21,22,23]. Methotrexate’s toxicity profile includes both acute and chronic effects. Acutely, it can cause mucositis, nausea, and hepatotoxicity. Chronic use can lead to hepatotoxicity, pulmonary fibrosis, and bone marrow suppression, which results in decreased blood cell counts, elevating the risk for infections, anemia, and bleeding. Due to its potential for liver damage and bone marrow suppression, regular monitoring is crucial for patients on methotrexate therapy. Additionally, it is teratogenic, hence contraindicated in pregnancy.

**Scheme 1:**
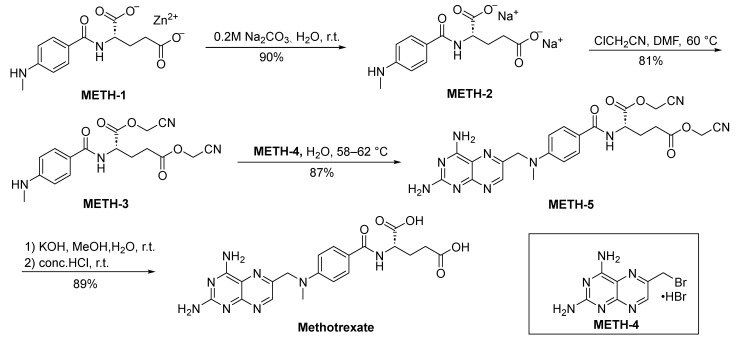
Synthesis of Methotrexate.

The synthesis of Methotrexate commences with the initial step, involving the treatment of METH-1 with a 0.2 M solution of sodium carbonate in water at room temperature, resulting in the formation of METH-2 (Scheme 1). Subsequently, METH-2 undergoes alkylation with chloroacetonitrile to yield METH-3 [24]. METH-3 is then further subjected to alkylation with METH-4, giving rise to METH-5. The ultimate transformation in this synthetic pathway involves the hydrolysis of METH-5, achieved through the utilization of potassium hydroxide in a mixture of methanol and water, followed by acidification with concentrated hydrochloric acid, ultimately leading to the successful production of Methotrexate.

#### 2.1.2. Gemcitabine

Gemcitabine (2′,2′-difluorodeoxycytidine) is a nucleoside analog that was first synthesized in the 1980s. In 1996, it received endorsement from the FDA and was subsequently marketed under the nomenclature Gemzar. This therapeutic agent has primarily been employed in the management of diverse carcinomas, encompassing breast, pancreatic, ovarian, and non-small cell lung cancer (NSCLC) [25]. Gemcitabine represents a highly potent and precise analog of deoxycytidine. Upon internalization within malignant cellular entities, Gemcitabine undergoes phosphorylation mediated by deoxycytidine kinase, ultimately yielding Gemcitabine monophosphate, which subsequently undergoes further conversion into its active derivatives, Gemcitabine diphosphate (dFdCDP) and Gemcitabine triphosphate (dFdCTP). These bioactive metabolites manifest as nucleoside analogs, thus engendering potent anti-tumorigenic effects. dFdCTP engages in a competitive interaction with deoxycytidine triphosphate (dCTP), vying for inclusion into the DNA structure, consequently engendering a competitive impediment to the process of DNA chain elongation. The placement of dFdCTP within the DNA chain at a non-terminal location obstructs its discernment and repair by the 3′5′-exonuclease enzyme responsible for proofreading. This phenomenon is denoted as “masked DNA chain termination”. The inclusion of dFdCTP into the DNA strand leads to eventual chain termination, provoking DNA fragmentation, and thereby inducing apoptotic cell demise in malignant cellular populations [26,27]. Gemcitabine possesses intrinsically self-potentiated pharmacological properties, which augment the likelihood of effective integration of Gemcitabine triphosphate into the DNA chain. This effect arises from the inhibitory influence of dFdCDP on ribonucleotide reductase, an enzyme pivotal in catalyzing reactions leading to the production of dCTP, a precursor for DNA synthesis. By diminishing dCTP levels, dFdCDP mitigates the competition encountered by Gemcitabine triphosphate for its incorporation into the DNA structure [28]. Furthermore, Gemcitabine has the capacity to attenuate the metabolic and eliminatory processes associated with the active metabolites within the target cellular milieu, thus extending the duration of elevated intracellular concentrations of these active metabolites. Gemcitabine is linked with hematological toxicities such as neutropenia, anemia, and thrombocytopenia. Non-hematological side effects include nausea, elevated liver enzymes, rash, and flu-like symptoms. A rare yet serious side effect is pulmonary toxicity, including interstitial pneumonitis.

**Scheme 2:**
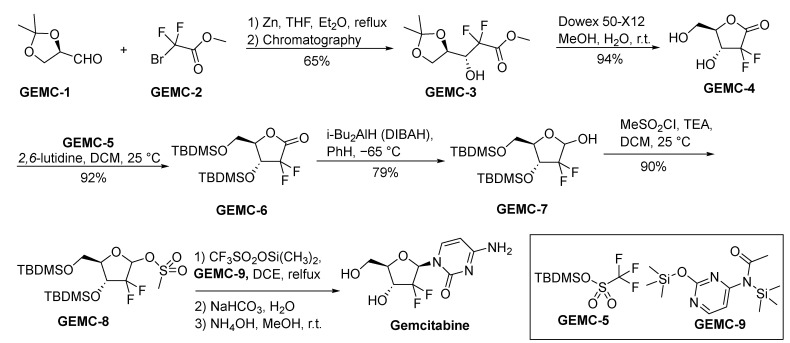
Synthesis of Gemcitabine.

The synthesis of Gemcitabine wis initiated by treating GEMC-2 with activated zinc (Zn) in an ether/THF solvent system (Scheme 2). Subsequent treatment with GEMC-1 yields a 3:1 mixture of diastereomers, which undergoes separation via HPLC, ultimately furnishing GEMC-3 [29]. The ensuing transformation involves the hydrolytic removal of isopropylidenes from GEMC-3, resulting in the formation of lactone GEMC-4. This lactone, GEMC-4, is silylated utilizing GEMC-5 in the presence of lutidine, affording the bis(tert-butyl dimethylsilyl) derivative, denoted as GEMC-6. Reduction of GEMC-6 with diisobutylaluminum hydride (DIBAH) yields the disilyl lactol GEMC-7. The reaction of the disilyl derivative, GEMC-7, with methanesulfonyl chloride leads to the formation of GEMC-8. The condensation of the mesylate GEMC-8 with GEMC-9, in the presence of trimethylsilyl triflate, in dichloroethane at reflux for 15 h produces a blocked nucleoside. Subsequently, deprotection of the nucleoside is accomplished via the hydrolytic removal of protecting groups, yielding Gemcitabine.

#### 2.1.3. Capecitabine

Capecitabine, formulated in the 1990s, represents an orally-delivered chemotherapeutic compound strategically conceived as a prodrug of 5-fluorouracil (5-FU). The rationale behind its development was to replicate the antineoplastic potency of 5-FU while affording the practicality associated with oral administration. In 1998, the FDA conferred approval to Capecitabine, subsequently commercialized under the appellation Xeloda. Initially sanctioned for the management of metastatic breast carcinoma, its therapeutic indications were later extended to encompass colorectal malignancies [30,31,32]. Capecitabine operates as a prodrug necessitating metabolic transformation, occurring primarily within the hepatic and neoplastic microenvironments, to generate its bioactive manifestation, namely, 5-FU. Within the confines of neoplastic cell populations, 5-FU undergoes a sequence of metabolic conversions culminating in the formation of two key metabolites: fluorodeoxyuridine monophosphate (FdUMP) and fluorouridine triphosphate (FUTP). FdUMP assumes a pivotal role by acting as an inhibitor of thymidylate synthase, an indispensable enzyme governing the process of DNA synthesis. This inhibition precipitates DNA damage, thus contributing to the therapeutic efficacy of 5-FU. On the other hand, FUTP gets incorporated into RNA, disrupting its function. Capecitabine has a toxicity profile that includes gastrointestinal effects such as diarrhea, nausea, and vomiting. A distinctive and noteworthy adverse event observed in some cases is the occurrence of hand–foot syndrome, also known as palmar–plantar erythrodysesthesia, which manifests as erythema, discomfort, and edema affecting the skin of the hands and the soles of the feet in afflicted patients. Other toxicities encompass cardiotoxicity, myelosuppression (reduced blood cell production), and hepatobiliary disorders. Given its potential toxicities, it is imperative for patients receiving Capecitabine to be monitored closely to manage and address any adverse reactions promptly [33,34].

**Scheme 3:**
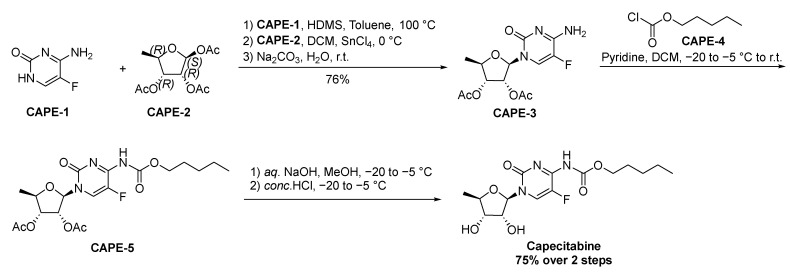
Synthesis of Capecitabine.

The synthetic process of Capecitabine commences with the treatment of CAPE-1 utilizing hexamethyldisilazane in toluene (Scheme 3). The resulting mixture is subsequently subjected to concentration under diminished pressure. Methylene chloride, CAPE-2, and anhydrous stannic chloride are then meticulously introduced dropwise into the reaction mixture [35]. Sodium bicarbonate is then introduced, followed by the gradual addition of water, culminating in the formation of CAPE-3. CAPE-3 is subsequently subjected to a condensation reaction with CAPE-4 in the presence of pyridine, conducted in dichloromethane, resulting in the production of CAPE-5. The final step in this synthetic route involves the hydrolysis of CAPE-5 with sodium hydroxide, followed by acidification with concentrated hydrochloric acid, ultimately yielding Capecitabine.

#### 2.1.4. Doxorubicin Hydrochloride

Doxorubicin Hydrochloride, an anthracycline antibiotic, was first isolated in the 1960s from the bacterium Streptomyces peucetius. Its antitumor activity was soon recognized, leading to its widespread adoption in oncology. Doxorubicin Hydrochloride received approval from the FDA in 1974 and was commercialized under the trade designation Adriamycin. It was sanctioned for therapeutic use in a spectrum of malignancies, encompassing, but not restricted to, breast cancer, ovarian cancer, lung cancer, gastric cancer, thyroid cancer, sarcoma, multiple myeloma, as well as pediatric malignancies [36,37]. Doxorubicin Hydrochloride has multifaceted mechanisms of action: (1) Intercalation into DNA: by inserting itself between base pairs, Doxorubicin disrupts the DNA’s structure and function; (2) Topoisomerase II Inhibition: it interferes with the action of topoisomerase II, an enzyme that helps relax supercoiled DNA. This interruption leads to DNA breaks and subsequently impedes replication and transcription; (3) Induction of reactive oxygen species (ROS): Doxorubicin initiates the generation of ROS, which have the potential to inflict harm upon cellular constituents, encompassing DNA, proteins, and lipids [38]. While Doxorubicin Hydrochloride is therapeutically beneficial, it presents significant toxicities. The most notorious is cardiotoxicity, which can lead to irreversible heart damage, including dilated cardiomyopathy. Other side effects include myelosuppression (reduced bone marrow activity), mucositis, alopecia, and nausea. The risk of cardiotoxicity, which may be dose-dependent, necessitates close monitoring of cumulative doses and cardiac function during treatment.

**Scheme 4:**
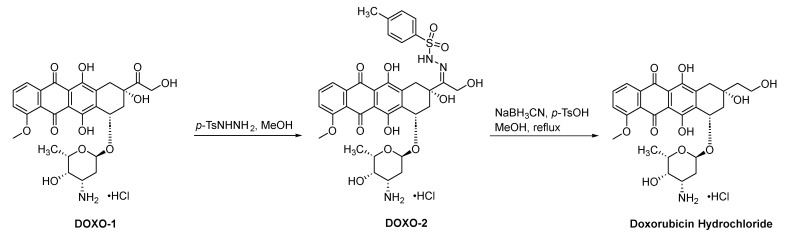
Synthesis of Doxorubicin Hydrochloride.

The synthesis of Doxorubicin Hydrochloride was initiated by converting DOXO-1 to DOXO-2 through treatment with p-toluenesulfonylhydrazide in a methanol medium, (Scheme 4) [39]. Subsequently, the reduction of DOXO-2 was achieved by employing sodium cyanoborohydride in the presence of p-toluenesulfonic acid, ultimately yielding the desired compound, Doxorubicin Hydrochloride.

#### 2.1.5. Epirubicin Hydrochloride

Epirubicin Hydrochloride, commercially known by various names including Ellence, garnered approval from the FDA in 1999, specifically for its application in adjuvant therapy for individuals afflicted with node-positive breast cancer [40]. Epirubicin Hydrochloride exhibits antimitotic and cytotoxic properties, exerting inhibitory effects on nucleic acid synthesis (both DNA and RNA) as well as protein synthesis through several mechanistic pathways that have been postulated. Epirubicin Hydrochloride engages in the formation of complexes with DNA through intercalation between base pairs. Furthermore, it exerts inhibitory effects on topoisomerase II activity by conferring stability to the DNA-topoisomerase II complex, thereby impeding the religation segment of the ligation-religation reaction catalyzed by topoisomerase II. Additionally, Epirubicin Hydrochloride disrupts DNA replication and transcription processes through the inhibition of DNA helicase activity [41]. In preclinical studies, Epirubicin Hydrochloride exhibited potent antitumor activity across various cancer cell lines. Its toxicity profile showed a lower cardiotoxic potential compared to its parent compound, Doxorubicin. Epirubicin Hydrochloride has exhibited remarkable therapeutic effectiveness, particularly among breast cancer patients, when administered both as a standalone therapeutic agent and in synergy with other chemotherapeutic compounds. It has established itself as a customary selection in the realm of adjuvant therapy for individuals afflicted with node-positive breast cancer. While Epirubicin Hydrochloride has a somewhat reduced cardiotoxicity profile compared to Doxorubicin, it can still cause cardiomyopathy. Other side effects include myelosuppression, alopecia, nausea, and mucositis [42,43].

**Scheme 5:**
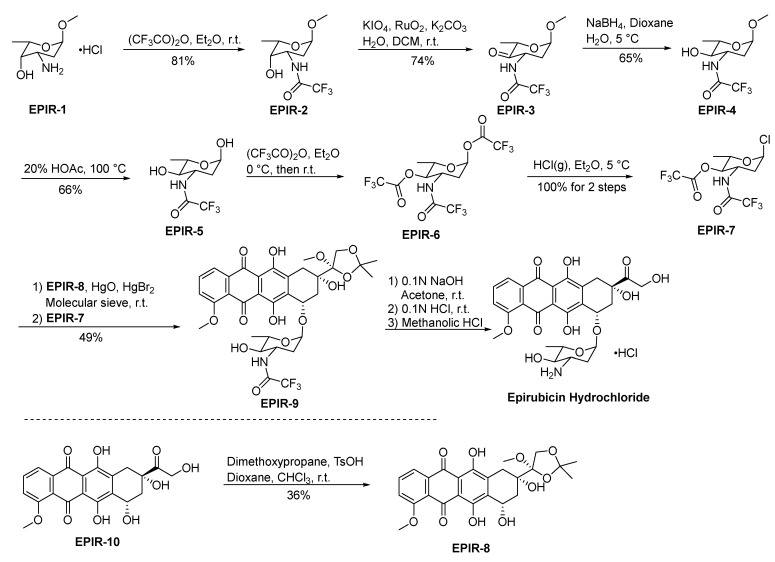
Synthesis of Epirubicin Hydrochloride.

The synthesis of Epirubicin Hydrochloride is initiated through the condensation of EPIR-1 with trifluoroacetic anhydride, yielding EPIR-2 (Scheme 5). Subsequent oxidation of EPIR-2 with a combination of KIO_4_, RuO_2_, and K_2_CO_3_ results in the formation of EPIR-3 [44]. EPIR-3 is subsequently reduced using NaBH_4_ to afford EPIR-4. The dealkylation of EPIR-4 is achieved through treatment with 20% HOAc at 100 °C, producing EPIR-5. EPIR-5 is esterified with trifluoroacetic anhydride, yielding EPIR-6, which is then regioselectively chlorinated in the presence of gaseous HCl to furnish EPIR-7. The regioselective alkylation of EPIR-8 with EPIR-7 carried out in the presence of HgO, HgBr_2_, and molecular sieve, generates EPIR-9. The final step in the synthesis involves the deprotection of EPIR-9 through treatment with 0.1 N NaOH, followed by further treatment with 0.1 N HCl and methanolic hydrochloric acid, ultimately yielding Epirubicin Hydrochloride. It is noteworthy that EPIR-8 is synthesized by treating EPIR-10 with dimethoxypropane and TsOH.

### 2.2. Progesterone Receptor Agonists

#### Megestrol Acetate

Megestrol Acetate stands as a synthetic analog of the natural steroid hormone progesterone, with pivotal applications in specific medical scenarios. Acquiring its seal of approval from the FDA in 1971, this drug is commercialized primarily under its trademark name, Megace, among others. The medicinal spectrum of Megestrol Acetate encompasses treatment for anorexia, cachexia, and unintentional weight loss, especially resonant in AIDS-afflicted patients. Furthermore, it serves as a palliative therapy for hormone-responsive breast and endometrial cancers [45,46,47]. Functioning as a progestin agonist, Megestrol Acetate commits to progesterone receptors. This binding instigates various therapeutic effects, including appetite stimulation and antagonism against particular hormone-sensitive malignancies. Animal studies spotlight its pronounced anti-gonadotropic and anti-estrogenic actions. Notably, it curtails the pituitary secretion of gonadotropins, instigating a drop in estrogen concentrations and exuding anti-estrogenic influences on target tissues. Patients under Megestrol Acetate therapy often experience enhanced appetite and weight uptick, marking its clinical significance. Potential adverse ramifications span thromboembolic events, fluid accumulation, hypertension, and adrenal suppression. Extended use may induce adrenal insufficiency [48].

**Scheme 6:**
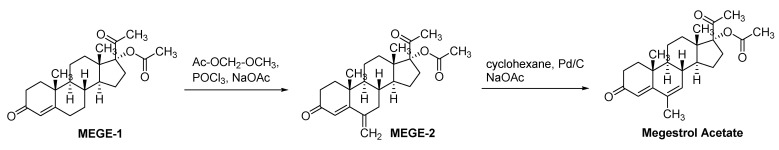
Synthesis of Megestrol Acetate.

The synthesis of Megestrol Acetate commences with the creation of a suspension, consisting of sodium acetate and distilled phosphorus oxychloride, which is refluxed in anhydrous chloroform at 70 °C for 30 min (Scheme 6). Following this, methoxymethyl acetate and MEGE-1 are introduced, and the resultant reaction solution is subjected to stirring for an additional 5 h at a temperature of 70 °C, culminating in the synthesis of MEGE-2. The synthesis culminates with the isomerization of MEGE-2, conducted in the presence of palladium on activated carbon (Pd/C) and sodium acetate in cyclohexane, ultimately affording Megestrol Acetate [49].

### 2.3. Aromatase Inhibitors

#### 2.3.1. Aminoglutethimide

Aminoglutethimide, a drug initially developed as an anticonvulsant, found its niche in the realm of endocrine therapy. Introduced and approved by the FDA in 1980 as an antiepileptic, it was soon recognized for its adrenal steroid synthesis inhibitory effects. Commercialized as Cytadren, this pharmaceutical agent has witnessed a progressive evolution in its clinical applications. It has notably demonstrated clinical utility in the management of Cushing’s syndrome and in the treatment of metastatic breast cancer in postmenopausal women [50]. Aminoglutethimide exerts inhibitory effects on adrenal steroidogenesis through the disruption of the enzymatic conversion of cholesterol into pregnenolone. This mechanism of action consequently leads to the suppression of the biosynthesis of a spectrum of steroid hormones, encompassing glucocorticoids, mineralocorticoids, estrogens, and androgens. In addition to adrenal inhibition, Aminoglutethimide can also inhibit the aromatase enzyme, which converts androgens to estrogens in peripheral tissues. This mechanism additionally enhances its anti-estrogenic attributes, rendering it a valuable therapeutic agent for managing hormone-sensitive breast carcinoma. Animal models highlighted Aminoglutethimide’s ability to significantly reduce plasma corticosterone and aldosterone levels, marking its potency in adrenal steroid synthesis inhibition. In clinical settings, the drug proved effective in diminishing cortisol production in Cushing’s syndrome patients and estrogen levels in postmenopausal breast cancer patients. Common side effects include dizziness, rash, and lethargy. More severe adverse reactions include hypothyroidism and agranulocytosis [51,52].

**Scheme 7:**
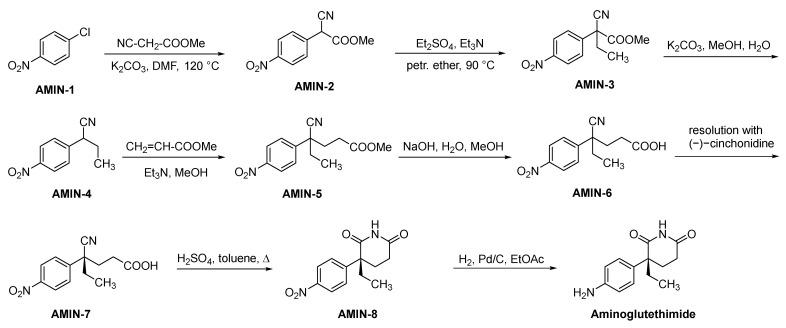
Synthesis of Aminoglutethimide.

The synthesis of Aminoglutethimide is initiated through a series of well-defined steps. It commences with the nucleophilic substitution of AMIN-1 with methyl cyanoacetate, yielding AMIN-2 (Scheme 7). Subsequently, AMIN-2 is subjected to alkylation with diethyl sulfate in the presence of triethylamine, giving rise to AMIN-3 [53]. The next step involves the hydrolysis and decarboxylation of the cyano ester AMIN-3, achieved by utilizing K_2_CO_3_ in MeOH, to produce AMIN-4. AMIN-4 undergoes a Michael addition reaction with methyl acrylate, forming the adduct AMIN-5. This adduct is subsequently hydrolyzed with NaOH, yielding the cyano acid AMIN-6, which is further resolved with (−)-cinchonidine, leading to the isolation of the desired (*R*)-enantiomer, AMIN-7. Acid-catalyzed cyclization of AMIN-7 in boiling toluene results in the formation of the glutarimide derivative, AMIN-8. The synthesis is brought to completion through the reduction of the nitro group in AMIN-8 to yield Aminoglutethimide, employing hydrogen gas over Pd/C.

#### 2.3.2. Anastrozole

The FDA approved Anastrozole in 1995, and it is commercially known under the trade name Arimidex. Predominantly employed in the context of adjuvant therapy, Anastrozole is indicated for administration to postmenopausal women afflicted with early-stage breast cancer characterized by the presence of hormone receptor-positive tumor profiles. Furthermore, it is designated for prescription as a first-line therapeutic option for postmenopausal women encountering locally advanced or metastatic breast cancer, in cases where the hormone receptor status is either positive or remains undetermined [54,55]. Anastrozole selectively inhibits the aromatase enzyme, responsible for converting androgens to estrogens in postmenopausal women. By blocking this conversion, estrogen levels decrease, inhibiting estrogen-sensitive breast cancer growth. Animal studies have demonstrated that Anastrozole effectively suppresses circulating estrogen levels, leading to a subsequent reduction in the size and growth of estrogen-dependent tumors. Clinically, Anastrozole has demonstrated a favorable profile in comparison to Tamoxifen, a selective estrogen receptor modulator, across various clinical trials. This superiority is evident in the extension of disease-free survival observed in patients with early-stage breast cancer. Potential side effects include hot flashes, joint symptoms, and potential reductions in bone mineral density leading to osteoporosis [56].

**Scheme 8:**
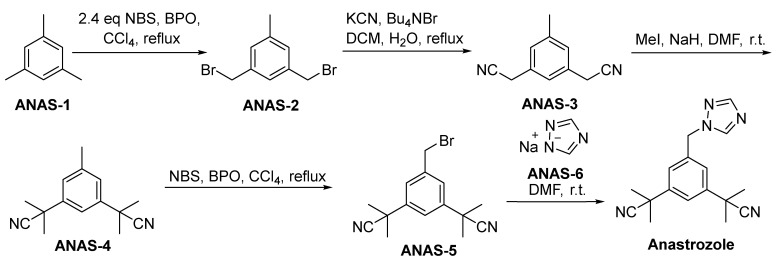
Synthesis of Anastrozole.

The synthesis of Anastrozole is initiated with the bromination of ANAS-1, using N-bromosuccinimide in the presence of benzoyl peroxide in refluxing CCl_4_ (Scheme 8). This reaction leads to the formation of ANAS-2 [57]. Subsequently, the bromine moiety in ANAS-2 is substituted with a cyano group, employing tetrabutylammonium bromide, resulting in the formation of ANAS-3. ANAS-3 undergoes alkylation with MeI to yield ANAS-4, followed by a second bromination step with NBS in the presence of BPO in refluxing CCl_4_, affording ANAS-5. The final step in this synthetic pathway involves the substitution of the bromine atom in ANAS-5 with ANAS-6, ultimately yielding Anastrozole.

#### 2.3.3. Letrozole

The FDA granted approval for Letrozole in 1997, with its commercialization occurring under the trade nomenclature Femara. Targeted primarily toward postmenopausal women, Letrozole plays a pivotal role in the therapeutic management of hormone receptor-positive early-stage breast cancer. Furthermore, it serves as the primary therapeutic option for individuals confronting advanced or metastatic breast cancer [58]. The mode of action of Letrozole involves the potent and sustained inhibition of the aromatase enzyme, a pivotal catalyst in the conversion of androgens to estrogens within postmenopausal subjects [59]. This culminates in diminished estrogen concentrations, obstructing the proliferation of estrogen-driven breast tumors. Animal studies have showcased Letrozole’s efficacy in markedly suppressing circulating estrogen levels, leading to a concomitant reduction in the magnitude and growth of estrogen-reliant malignancies. Clinical trials elucidate Letrozole’s superiority over Tamoxifen, underscoring an enhanced disease-free survival in early breast cancer cohorts. Adverse effects span from hot flashes and fatigue to potential osteoporosis due to decreased bone mineral density [60].

**Scheme 9:**
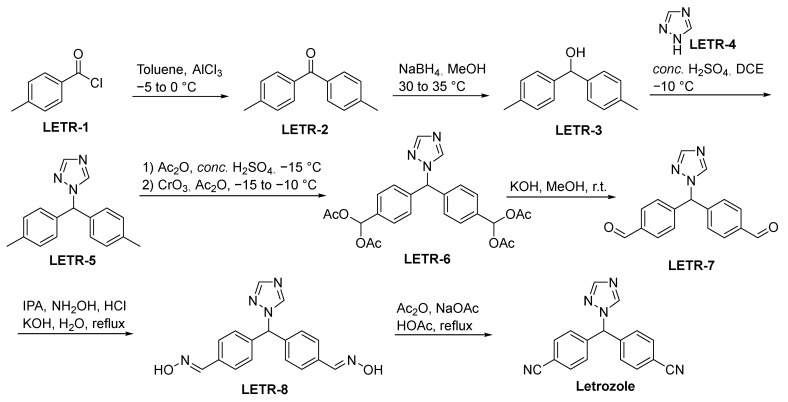
Synthesis of Letrozole.

The synthesis of Letrozole commences with the treatment of toluene with LETR-1 at a temperature range of −5 to 0 °C, resulting in the formation of LETR-2 (Scheme 9). Subsequent reduction of LETR-2 is carried out with NaBH_4_, yielding LETR-3 [61]. LETR-3 is subjected to a reaction with LETR-4 in the presence of concentrated H_2_SO_4_ in 1,2-dichloroethane (DCE), giving rise to LETR-5. The synthetic process proceeds with the oxidation of LETR-5 utilizing CrO_3_ in conjunction with acetic anhydride and concentrated sulfuric acid, resulting in the synthesis of LETR-6. Subsequently, LETR-6 is treated with KOH in MeOH, affording LETR-7. The transformation of LETR-7 is then achieved through a reaction with hydroxylamine hydrochloride, ultimately yielding LETR-8. The final step in the synthesis involves the treatment of LETR-8 with acetic anhydride and sodium acetate in acetic acid, resulting in the production of Letrozole.

#### 2.3.4. Exemestane

Securing its FDA approval in 1999, Exemestane is commercially dispensed under the trade name Aromasin. Primarily intended for administration to postmenopausal women, Exemestane assumes a pivotal role in the management of hormone receptor-positive early-stage breast cancer, particularly in the context of adjuvant therapy following a regimen of two to three years of Tamoxifen treatment. It is also pivotal in cases of advanced breast cancer post Tamoxifen failure [62]. In contrast to nonsteroidal aromatase inhibitors, Exemestane represents a steroidal compound that engages in irreversible binding with and inactivation of the aromatase enzyme. This action effectively impedes the enzymatic conversion of androgens into estrogens, particularly in postmenopausal female individuals [63]. This leads to notable estrogen reduction, impeding estrogen-sensitive breast cancer growth. Animal studies accentuated Exemestane’s potency in considerably lowering circulating estrogen concentrations, consequently mitigating estrogen-dependent tumor proliferation. Clinical trials have spotlighted Exemestane’s effectiveness, especially when juxtaposed with Tamoxifen, marking a pronounced upswing in disease-free survival among early breast cancer patients. Side effects might encompass hot flashes, fatigue, and osteoporosis stemming from a dip in bone mineral density [64].

**Scheme 10:**
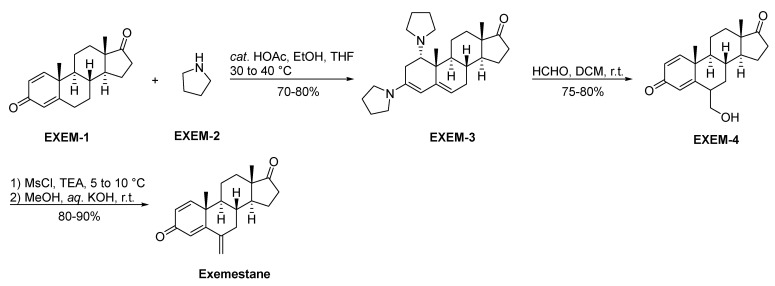
Synthesis of Exemestane.

The synthetic process of Exemestane is initiated through the reaction of EXEM-1 and EXEM-2 in the presence of acetic acid in a mixture of denatured ethanol and THF, maintaining a temperature range between 30 and 40 °C for a minimum duration of 16 h, culminating in the synthesis of EXEM-3 (Scheme 10). This intermediate subsequently undergoes a reaction with HCHO at room temperature in dichloromethane, yielding EXEM-4. EXEM-4 is further subjected to treatment with methylsulfonyl chloride (MsCl) and triethylamine (TEA), followed by subsequent treatment with aqueous KOH in methanol. This strategic sequence of reactions ultimately results in the synthesis of Exemestane [65].

### 2.4. Selective Estrogen Receptor Modulators (SERMs)

#### 2.4.1. Tamoxifen Citrate

Tamoxifen Citrate, a foremost contender in the domain of breast cancer therapeutics, is classified within the selective estrogen receptor modulator (SERM) category. The FDA granted its approval for Tamoxifen in 1977, and it is prominently marketed under the trade designation Nolvadex. Tamoxifen serves as the preferred therapeutic choice for both pre- and postmenopausal women who have been diagnosed with early or metastatic breast cancer characterized by the presence of hormone receptor positivity. Additionally, it finds application as a prophylactic measure for women identified as having a heightened susceptibility to the development of this malignancy [66]. Tamoxifen engages in interactions with estrogen receptors, demonstrating a paradoxical profile. While it functions as an estrogen antagonist in breast tissues, it can paradoxically exhibit estrogen agonistic activity in other anatomical sites, such as the endometrium [67]. This duality stalls the growth of estrogen-sensitive breast tumors. Animal models unveiled Tamoxifen’s ability to hinder the growth of mammary tumors, owing to its antiestrogenic properties [68]. Several landmark trials, including the National Surgical Adjuvant Breast and Bowel Project (NSABP) studies, emphasized Tamoxifen’s pivotal role in reducing recurrence and boosting survival in early breast cancer cases. Potential adverse effects range from menopausal symptoms to more grave ones like endometrial cancer and thromboembolic events [69].

**Scheme 11:**
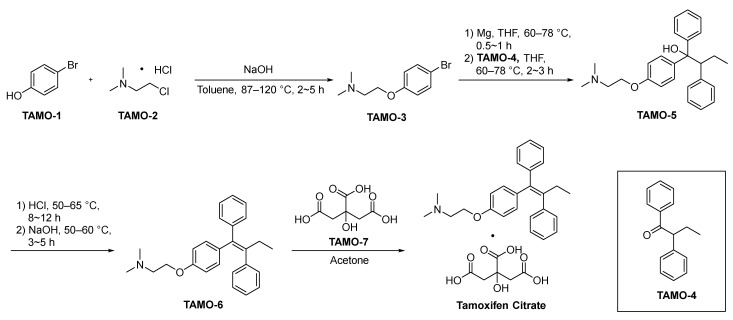
Synthesis of Tamoxifen Citrate.

The synthetic process of Tamoxifen Citrate is initiated through the etherification of TAMO-1 and TAMO-2, employing NaOH as a reagent. This reaction leads to the formation of TAMO-3 (Scheme 11). Subsequently, a Grignard reaction between TAMO-3 and TAMO-4 furnishes TAMO-5. TAMO-5 is then dehydrated in the presence of HCl and subsequently alkalized with NaOH, yielding TAMO-6. The synthesis is finalized by treating TAMO-5 with TAMO-7, ultimately yielding Tamoxifen Citrate [70].

#### 2.4.2. Toremifene

The U.S. FDA granted Toremifene its approval in 1997, marketing it with the trade name Fareston. Toremifene is prescribed predominantly for postmenopausal women bearing metastatic breast cancer characterized as estrogen receptor-positive [71]. Toremifene falls under the SERM category. Like other SERMs, Toremifene engages with estrogen receptors. Acting as an antagonist in breast tissues, it effectively thwarts estrogen-mediated tumor growth, while in other tissues, its activity can be agonistic. It exhibits agonistic estrogenic activity with notable implications for the cardiovascular and osseous systems, while demonstrating comparatively subdued estrogenic effects on uterine tissue [72]. In animal studies, Toremifene showcased its prowess by substantially mitigating the growth of estrogen-reliant mammary tumors [73]. Clinical evaluations have pinpointed Toremifene’s efficacy, noting its comparable performance to Tamoxifen, especially in metastatic breast cancer contexts. Potential side effects envelop menopausal symptoms, sweating, and nausea. Rarer, yet significant concerns include thromboembolic events and potential endometrial changes [74].

**Scheme 12:**
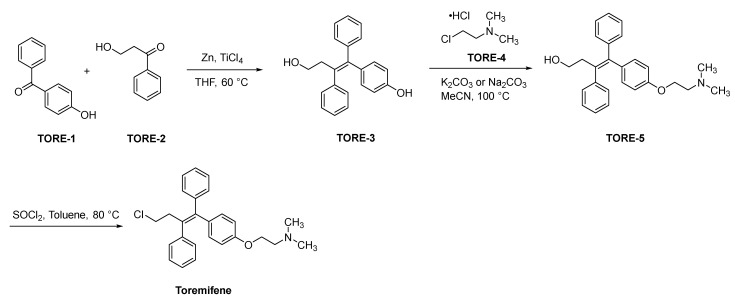
Synthesis of Toremifene.

The synthesis of Toremifene commences with a McMurry reaction, wherein TORE-1 is reacted with TORE-2 in the presence of Zn and TiCl_4_, ultimately yielding TORE-3 (Scheme 12) [75]. Subsequently, TORE-3 undergoes a selective alkylation of the phenol hydroxyl group, employing the hydrochloride of TORE-4, resulting in the formation of TORE-5. The synthesis is concluded by treating TORE-5 with dichlorosulfoxide, ultimately affording Toremifene.

#### 2.4.3. Raloxifene

The therapeutic trajectory of Raloxifene commenced in 1997 with its endorsement by the FDA for clinical utilization, and it made its mark in the pharmaceutical market under the trade name Evista. While the principal indication for Raloxifene revolves around its approval for the prophylaxis and therapeutic intervention of osteoporosis among postmenopausal women, its therapeutic advantages extend beyond this scope. This pharmaceutical agent additionally functions to attenuate the susceptibility to invasive breast carcinoma among postmenopausal women afflicted with osteoporosis or those exhibiting an elevated predisposition to such malignancies [76]. Raloxifene’s core target revolves around the estrogen receptor. It showcases duality: antagonizing estrogen in breast and endometrial tissues, yet championing its positive impacts on bone in osteoporotic settings [77]. Animal models have illuminated Raloxifene’s unique attributes. It not only amplifies bone mineral density but achieves this without hyperstimulating the endometrial lining, setting it apart from traditional estrogens [78]. In human trials, Raloxifene’s efficacy emerges prominently, enhancing bone density and curtailing fracture risks in postmenopausal subjects. Although predominantly well-tolerated, Raloxifene is not exempt from adverse effects, with commonly encountered manifestations encompassing hot flashes and musculoskeletal discomfort, such as leg cramps. However, clinicians remain vigilant about its association with increased thromboembolic events [79].

**Scheme 13:**
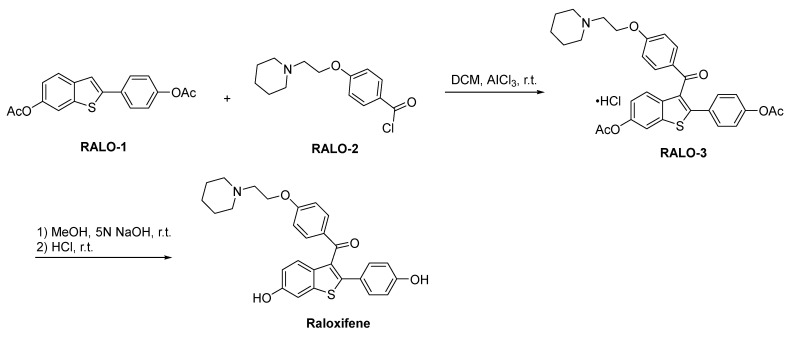
Synthesis of Raloxifene.

The synthesis of Raloxifene commences with the Friedel–Crafts acylation of RALO-1 using RALO-2 in the presence of AlCl_3_, yielding the resultant compound RALO-3 (Scheme 13). Subsequently, RALO-3 is subjected to hydrolysis with 5N NaOH, followed by acidification with HCl, ultimately yielding Raloxifene [80].

### 2.5. Selective Estrogen Receptor Degraders (SERDs)

#### 2.5.1. Fulvestrant

Fulvestrant’s inception into clinical use was solidified in 2002 when the FDA granted its approval. Commercially known as Faslodex, its introduction marked a new chapter in hormone therapy. The primary therapeutic indication for Fulvestrant lies in the management of hormone receptor-positive metastatic breast carcinoma among postmenopausal women, particularly those who have experienced disease progression subsequent to anti-estrogen therapy [81]. Unlike traditional SERMs, Fulvestrant directly targets and degrades the estrogen receptor. It functions as an unadulterated antagonist of the estrogen receptor, binding to the receptor, and blocking and accelerating its degradation. Animal models have reflected the drug’s prowess. Fulvestrant demonstrated an inhibitory effect on the proliferation of breast cancer cells resistant to Tamoxifen, heralding a potential breakthrough for treatment-resistant cases [82]. In head-to-head trials against advanced aromatase inhibitors, Fulvestrant demonstrated equivalent, if not superior, efficacy, especially in patients with prior endocrine therapy. Most patients tolerate Fulvestrant well. Nonetheless, frequently encountered adverse events encompass local reactions at the injection site, sensations of nausea, and manifestations of fatigue. More serious events, although rare, involve hepatic impairment [83].

**Scheme 14:**
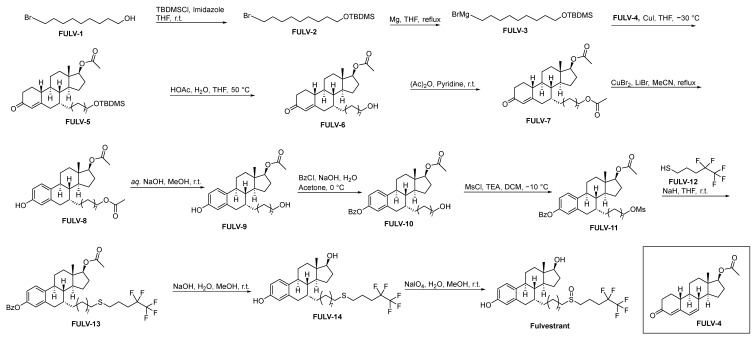
Synthesis of Fulvestrant.

The synthesis of Fulvestrant is initiated with the protection of FULV-1 using tert-butyldimethylsilyl chloride and imidazole in THF, yielding FULV-2 (Scheme 14) [84]. Subsequently, FULV-2 undergoes a reaction with Mg in the same solvent, leading to the formation of FULV-3. The synthesis continues with the reaction of FULV-3 with CuI, resulting in the formation of the corresponding organocuprate that subsequently condenses with FULV-4, yielding FULV-5. The silyl ether group of FULV-5 is cleaved using HOAc and water in THF, affording alcohol FULV-6, which is esterified with Ac_2_O and pyridine, producing the corresponding diacetate, FULV-7. The synthesis then involves the aromatization of the enone FULV-7 using CuBr_2_ and LiBr in refluxing acetonitrile, yielding the phenol FULV-8. This compound is selectively hydrolyzed with NaOH in methanol, providing the primary alcohol, FULV-9. The selective esterification of the phenolic hydroxyl group of FULV-9 with benzoyl chloride and NaOH in a mixture of acetone and water-furnished FULV-10. FULV-10, through a reaction of its primary hydroxyl group with mesyl chloride and TEA in dichloromethane, leads to the formation of FULV-11. Compound FULV-11 is then condensed with FULV-12, employing NaH in THF, resulting in the production of the thioether, FULV-13. FULV-13 is subjected to basic hydrolysis of the ester groups using NaOH in a mixture of methanol and water, yielding the corresponding dihydroxy compound, FULV-14. The synthesis culminates in the oxidation of FULV-14 with sodium metaperiodate to provide Fulvestrant.

#### 2.5.2. Elacestrant

Elacestrant is classified as a non-steroidal small molecule serving as an antagonist of the estrogen receptor (ER) [85]. In 2023, regulatory approval from the FDA was granted, leading to its commercialization under the trade designation Orserdu. This approval pertains to its utilization in the management of advanced or metastatic breast cancer characterized by ER−positive (ER+), human epidermal growth factor receptor 2-negative (HER2−), and estrogen receptor 1 gene (ESR1) mutations. Elacestrant represents an orally-administered SERD exhibiting affinity for the estrogen receptor-alpha (ERα) [86]. Tumors of the breast exhibiting the expression of ERα are reliant upon estrogen-induced proliferative signaling pathways. As a consequence, therapeutic strategies directed towards the ER are widely employed in the management of this particular malignancy. SERDs constitute a class of endocrine therapies designed to oppose the transcriptional functionality of the ER while facilitating its proteolytic degradation. Within breast cancer cells characterized by ER+, HER2−, Elacestrant exhibits the capacity to impede cell proliferation driven by 17β-estradiol and initiates degradation of ERα via the proteasomal pathway. Elacestrant additionally retards the nuclear translocation of ER and facilitates ER turnover, thereby perturbing subsequent intracellular signaling cascades [87]. In both in vitro and in vivo settings, Elacestrant demonstrates anti-tumor efficacy against breast cancer models characterized by ER+, HER2−profiles, which exhibit resistance to Fulvestrant and cyclin-dependent kinase 4/6 inhibitors. Furthermore, Elacestrant exhibits activity in cancer models harboring mutations in the ESR1 [88,89]. Elacestrant has demonstrated a predominantly positive safety profile in the context of clinical trials. Frequently encountered adverse events encompass symptoms such as hot flashes, nausea, and fatigue, which are generally amenable to management.

**Scheme 15:**
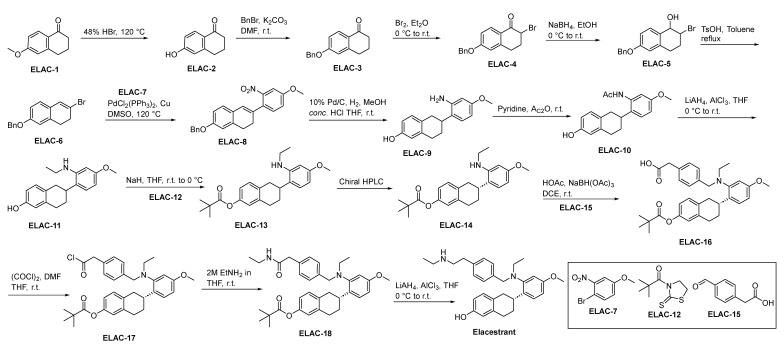
Synthesis of Elacestrant.

The synthesis of Elacestrant is initiated by demethylating ELAC-1 in the presence of 48% HBr at a temperature of 120 °C, resulting in the formation of ELAC-2 (Scheme 15). This intermediate is then treated with (bromomethyl)benzene and potassium carbonate, leading to the generation of ELAC-3 [90]. Subsequent bromination of ELAC-3 using Br_2_ gives rise to ELAC-4. ELAC-4 is subsequently reduced with sodium borohydride to yield ELAC-5, followed by treatment with p-toluenesulfonic acid and refluxing in toluene, affording ELAC-6. The synthesis proceedes with the coupling of ELAC-6 and ELAC-7 in the presence of palladium dichloride-bis(triphenylphosphine) and copper (PdCl_2_(PPh_3_)_2_, Cu) in DMSO at 120 °C, resulting in the formation of ELAC-8. Subsequent reduction of ELAC-8 in the presence of Pd/C affords ELAC-9, which is treated with acetic anhydride in pyridine to give ELAC-10. ELAC-10 undergoes reduction with lithium aluminum hydride in the presence of aluminum trichloride, providing ELAC-11, which is then esterified with ELAC-12, yielding ELAC-13. The chiral separation of ELAC-13 leads to the isolation of ELAC-14. Reductive amination of ELAC-14 with ELAC-15 in the presence of NaBH(OAc)_3_ results in the formation of ELAC-16, which is then treated with oxalyl chloride and DMF to afford ELAC-17. ELAC-17 is stirred in a 2 M solution of ethylamine in ethanol, giving rise to ELAC-18. The synthesis is completed with the reduction of ELAC-18 using lithium aluminum hydride in the presence of aluminum trichloride, ultimately yielding Elacestrant.

### 2.6. Radioactive Diagnostic Agent

#### Fluoroestradiol F-18

Fluoroestradiol F-18, commercially recognized as Detectnet, was granted approval by the FDA in 2020. This groundbreaking radiopharmaceutical offers an innovative diagnostic edge, specifically formulated for positron emission tomography (PET) imaging for patients diagnosed with ER+ breast cancer. Its approval heralds a new chapter in diagnostic imaging, enabling precise assessment of the ER status of breast lesions, both during initial disease staging and during subsequent evaluations. The principal target activity of Fluoroestradiol F-18 is the estrogen receptor, given its intrinsic design as a radiolabeled analog of estradiol, ensuring its selective affinity for ERs [91]. Mechanistically, Fluoroestradiol F-18 operates by mimicking estradiol. Post-administration, it exhibits selective binding to ERs, particularly prevalent in cancerous cells. Integral to its function, the F-18 isotope radiates positrons, subsequently detected via PET scans, illuminating ER-abundant areas or malignant tumors [92]. In preclinical pharmacodynamics investigations, the specificity of Fluoroestradiol F-18 for ERs was established, emphasizing its capability to differentiate ER+ malignancies from their ER−counterparts. From a clinical efficacy perspective, the imaging potential of Fluoroestradiol F-18 has been exemplified in its significant correlation with biopsy-ascertained ER status, offering a non-invasive instrument for ER status determination in metastatic lesions. Addressing toxicity, the drug’s profile remains largely benign, with primary apprehensions circumscribed to radiation exposure—a generic concern for all radiopharmaceuticals. Notably, its clinical application has rarely seen significant adverse reactions [93].

**Scheme 16:**
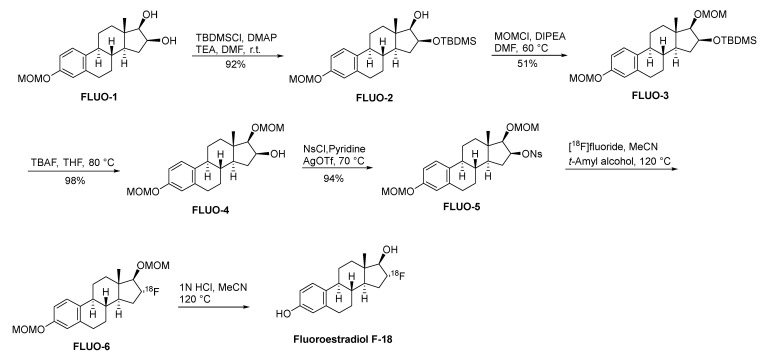
Synthesis of Fluoroestradiol F-18.

The synthesis of Fluoroestradiol F-18 is initiated with the selective silylation of FLUO-1, utilizing t-butyldiphenylsilyl chloride, yielding FLUO-2, followed by the protection of FLUO-2 using methoxymethyl chloride (MOMCl) to produce FLUO-3 (Scheme 16). Subsequently, the TBDMS group is removed by treating FLUO-3 with tetrabutylammonium fluoride (TBAF), resulting in the formation of FLUO-4. FLUO-5 is synthesized from FLUO-4 using *p*-nitrobenzenesulfonyl chloride (NsCl) [94]. The [^18^F] fluorination is carried out by employing FLUO-5 and [^18^F] fluoride in a mixture of acetonitrile and tert-amyl alcohol. Subsequent to the chemical reaction, solvent evaporation ensues, and the resultant residue is reconstituted in acetonitrile. An aqueous solution of hydrochloric acid is introduced, thereby yielding Fluoroestradiol F-18.

### 2.7. HER2 Receptor Tyrosine Kinase Inhibitors

#### 2.7.1. Lapatinib Ditosylate

Lapatinib Ditosylate, commercialized as Tykerb, was given the green light by the FDA in 2007. This innovative therapeutic compound occupies a unique position within the therapeutic landscape for metastatic breast cancer characterized by HER2-positive status, particularly in individuals who have previously undergone Trastuzumab therapy. Lapatinib’s primary indication zeroes in on HER2-positive metastatic breast cancers. Generally co-administered with Capecitabine, it provides a therapeutic alternative for cases that have proven Trastuzumab-resistant [95]. In the realm of drug target activity, Lapatinib stands out with its bifunctional prowess. It selectively engages with both HER2 and epidermal growth factor receptor (EGFR) tyrosine kinases, pivotal players in the unfolding narrative of breast cancer advancement. At the core of its mode of action, Lapatinib exhibits preferential binding to the ATP-binding domains of these kinase enzymes. This binding puts a halt to downstream signaling pathways, effectively thwarting tumor cell proliferation [96]. Preclinical pharmacodynamics studies painted a promising picture, with Lapatinib effectively halting tumor growth in HER2-overexpressing xenograft models. On the clinical efficacy front, Lapatinib has been extensively recorded, particularly in combination with chemotherapeutic agents, for its capacity to enhance progression-free survival metrics within the cohort of patients afflicted by metastatic breast cancer characterized by HER2-positive status. However, Lapatinib’s toxicity includes diarrhea, dermatological manifestations, and occasional perturbations in liver function [97].

**Scheme 17:**
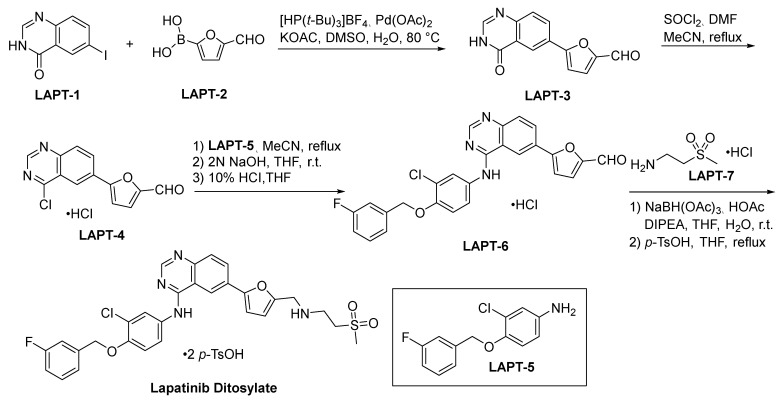
Synthesis of Lapatinib Ditosylate.

The synthesis of Lapatinib Ditosylate is initiated through a Suzuki coupling reaction between LAPT-1 and LAPT-2, resulting in the formation of LAPT-3 (Scheme 17). This is followed by the chlorination of LAPT-3 using SOCl_2_ and DMF in refluxing MeCN, leading to the generation of LAPT-4 [98]. Subsequently, the chlorine atom in LAPT-4 is displaced with LAPT-5, yielding LAPT-6. The final step in the synthesis involves the reductive amination of LAPT-6 with LAPT-7, followed by treatment with *p*-TsOH, ultimately resulting in the formation of Lapatinib Ditosylate.

#### 2.7.2. Neratinib

Neratinib, commercialized under the trade name Nerlynx, earned its commendation from the FDA in 2017. Emerging as a formidable therapeutic candidate, it is precisely designed for individuals confronting early-stage breast cancer characterized by overexpression or amplification of the HER2 receptor. The primary clinical application of Neratinib lies in its role as an extended adjuvant therapy for adult patients who have previously undergone Trastuzumab-based treatment for early-stage breast cancer characterized by HER2-positive status. Probing into its drug target activity, Neratinib stands out as an irreversible tyrosine kinase inhibitor (TKI) addressing several kinases, most notably HER1 (EGFR), HER2, and HER4. Its mechanism of action is profound. Neratinib establishes a covalent interaction with specific kinase domains, subsequently inhibiting their phosphorylation and activation. As a result, a significant modulation in downstream signaling pathways is observed, leading to a reduction in cellular proliferation and viability [99]. Preclinical pharmacodynamics studies illuminated Neratinib’s capability in antagonizing HER2-amplified cells. Key observations included a remarkable tumor growth restraint in tumor xenograft models [100]. Concerning clinical efficacy, research pillars like the ExteNET trial exemplified Neratinib’s prowess in diminishing the risk of invasive disease recurrence following adjuvant trastuzumab therapy. However, Neratinib’s journey is not devoid of toxicity concerns. Predominant side effects encompass diarrhea, hepatotoxicity, and rashes, underscoring the importance of patient monitoring during therapy [101].

**Scheme 18:**
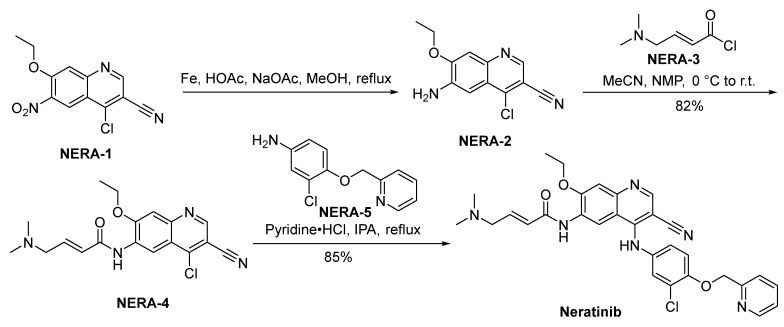
Synthesis of Neratinib.

The synthesis of Neratinib commences with the reduction of NERA-1 using iron, resulting in the formation of NERA-2 (Scheme 18). Subsequently, NERA-2 undergoes condensation with NERA-3, leading to the production of NERA-4. The chloride group in NERA-4 is then displaced with NERA-5 in a solvent mixture of pyridine and isopropanol (IPA), ultimately yielding Neratinib [102].

#### 2.7.3. Tucatinib

Tucatinib, commercialized under the trade designation Tukysa, received approval from the FDA in 2020. Tucatinib is prescribed in conjunction with Trastuzumab and Capecitabine for the management of advanced, non-surgically resectable, or metastatic HER2-positive breast cancer in adult patients, encompassing those with brain metastases, who have undergone one or multiple prior regimens involving anti-HER2 therapies within the metastatic context. Tucatinib represents a kinase inhibitor characterized by its pronounced selectivity towards HER2. Its mechanism of action entails selective binding to and subsequent inhibition of HER2 activity, thereby impeding the proliferation of tumor cells expressing HER2 [103]. Preclinical investigations revealed the antineoplastic potential of Tucatinib within breast cancer models characterized by HER2 overexpression, with augmented antineoplastic efficacy observed when administered in conjunction with Trastuzumab or Capecitabine [104]. The clinical effectiveness of Tucatinib underwent assessment within the pivotal HER2CLIMB trial, wherein a substantial enhancement in both progression-free survival and overall survival was observed in patients subjected to the Tucatinib, Trastuzumab, and Capecitabine combination regimen in comparison to those receiving Trastuzumab and Capecitabine as monotherapies [105]. Frequently encountered adverse events encompass gastrointestinal disturbances, hand–foot syndrome, emesis, fatigue, hepatotoxic manifestations, and cutaneous eruptions. Serious side effects can include severe diarrhea and liver problems.

**Scheme 19:**
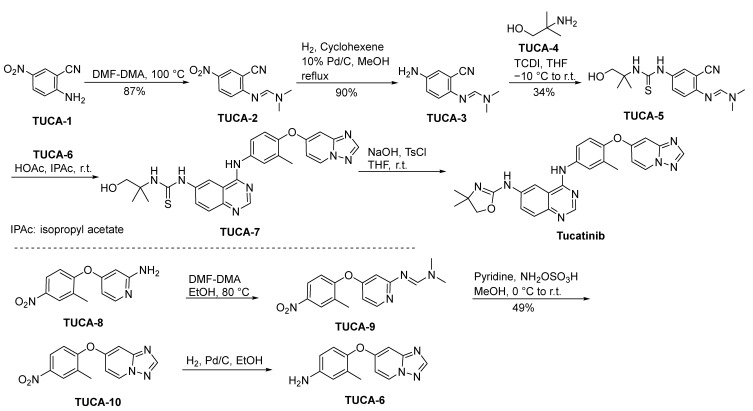
Synthesis of Tucatinib.

The synthesis of Tucatinib is initiated with the treatment of TUCA-1 using dimethoxy-N,N-dimethylmethanamine (DMF-DMA) at a temperature of 100 °C for a duration of 2 h, culminating in the generation of TUCA-2 (Scheme 19). Subsequently, TUCA-2 undergoes reduction in the presence of 10% Pd/C in methanol, leading to the generation of TUCA-3 [106]. TUCA-3 is then treated with thiocarbonyldiimidazole and TUCA-4, yielding TUCA-5. The synthesis proceeds with the cyclization of TUCA-5 using TUCA-6, ultimately forming TUCA-7. Finally, TUCA-7 is subjected to treatment with NaOH and tosyl chloride, ultimately giving Tucatinib. The synthesis of TUCA-6 began with the treatment of TUCA-8 using DMF-DMA in ethanol at 80 °C for 1 h, leading to the formation of TUCA-9. Subsequently, TUCA-9 undergoes cyclization in the presence of pyridine and hydroxylamine sulfonic acid in methanol, resulting in TUCA-10. The synthesis is finalized with the reduction of TUCA-10 in the presence of Pd/C in ethanol, affording TUCA-6.

### 2.8. Cyclin-Dependent Kinases 4 and 6 (CDK4/6) Inhibitors

#### 2.8.1. Palbociclib Hydrochloride

Palbociclib Hydrochloride, marketed under the trade designation Ibrance, received accelerated approval from the FDA in 2015. Palbociclib is authorized for utilization in conjunction with select endocrine therapies for managing advanced or metastatic breast cancer characterized by hormone receptor (HR)-positive, HER2-negative status, applicable to both postmenopausal women and men. Palbociclib functions as a discerning inhibitor of CDK4 and CDK6, thereby impeding the transition of the cell cycle from the G1 phase to the S phase, consequently suppressing the proliferation of neoplastic cells [107]. Preclinical investigations demonstrated the capability of Palbociclib to impede the proliferation of in vitro ER-positive breast cancer cell lines, alongside its efficacy in diminishing tumor growth as evidenced in xenograft models [108]. The authorization of Ibrance was rooted in the findings of the PALOMA-1/TRIO-18 clinical trial, which elucidated a notable enhancement in progression-free survival (PFS) among patients administered Palbociclib in conjunction with Letrozole, in comparison to those subjected to Letrozole monotherapy [109]. Frequently encountered adverse events comprise neutropenia, leukopenia, fatigue, anemia, and upper respiratory tract infections. Serious complications might arise from prolonged neutropenia.

**Scheme 20:**
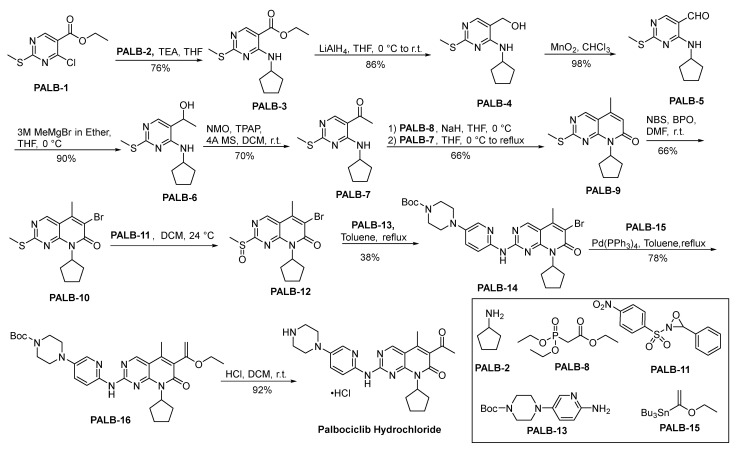
Synthesis of Palbociclib Hydrochloride.

The synthesis of Palbociclib Hydrochloride is initiated with the displacement of the chlorine atom in PALB-1 using PALB-2, resulting in the formation of PALB-3 (Scheme 20) [110]. The ester moiety present in PALB-3 undergoes a conversion process into an aldehyde via a two-stage reduction–oxidation protocol. This entails initial treatment with lithium aluminum hydride, succeeded by subsequent oxidation employing manganese (IV) oxide, ultimately furnishing aldehyde PALB-5. Subsequently, PALB-5 undergoes treatment with methylmagnesium bromide, leading to the formation of PALB-6, which is further oxidized to ketone PALB-7. The oxidation procedure is executed utilizing a choice of either catalytic tetra-n-propylammonium perruthenate or N-methylmorpholine N-oxide. Ketone PALB-7 is then subjected to a reaction with PALB-8 and sodium hydride in THF, resulting in the production of PALB-9. Treatment of PALB-9 with N-bromosuccinimide yields PALB-10. The oxidation of PALB-10 in the presence of PALB-11 affords PALB-12, which is subsequently substituted with PALB-13, leading to the formation of PALB-14. The synthesis is continued with the Stille coupling of PALB-14 and PALB-15, ultimately resulting in the formation of PALB-16. Finally, the deprotection of PALB-16 using hydrogen chloride gas (HCl) affords Palbociclib Hydrochloride.

#### 2.8.2. Ribociclib

Ribociclib, commercialized under the trade designation Kisqali, received regulatory endorsement from the FDA in 2017. Ribociclib is authorized for utilization in conjunction with an aromatase inhibitor as the primary endocrine-based treatment option for postmenopausal women afflicted by advanced or metastatic breast cancer characterized by HR-positive, HER2-negative status. Ribociclib serves as a discerning inhibitor targeting CDK4 and CDK6, thereby arresting the cell cycle transition from the G1 phase to the S phase, subsequently curtailing the proliferation of neoplastic cells [111]. Preclinical findings demonstrated that Ribociclib attenuated the proliferation of breast cancer cell lines through the interruption of cell cycle progression, culminating in the induction of senescence and apoptosis [112]. The MONALEESA-2 trial established the efficacy of Ribociclib. A marked enhancement in PFS was evident among postmenopausal women afflicted by HR-positive and HER2-negative advanced breast cancer when subjected to Ribociclib in conjunction with Letrozole, as compared to those who underwent Letrozole monotherapy [113]. Frequently encountered adverse events encompass neutropenia, nausea, infections, fatigue, and diarrhea. Noteworthy adverse reactions may encompass hepatic disorders, prolonged QT interval, and severe neutropenia.

**Scheme 21:**
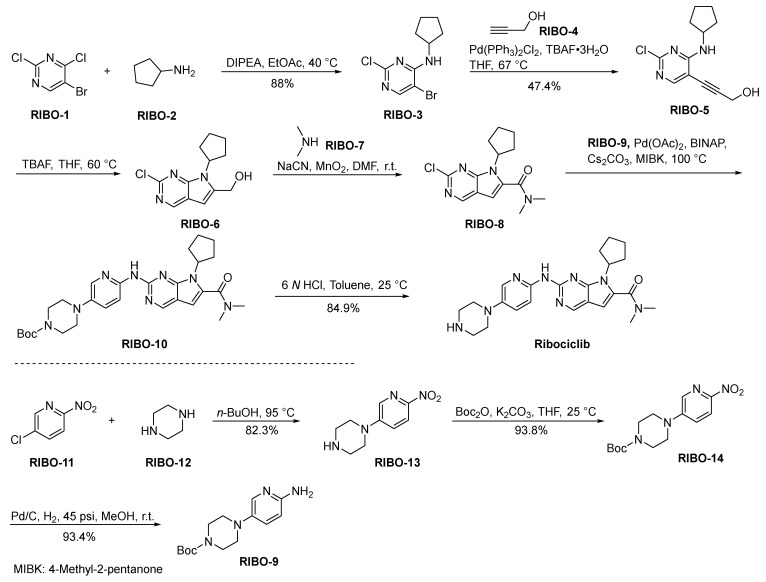
Synthesis of Ribociclib.

The synthesis of Ribociclib is initiated with the substitution of RIBO-1 with RIBO-2 in the presence of DIPEA, yielding RIBO-3 (Scheme 21) [114]. Subsequently, a Sonogashira coupling between RIBO-3 and RIBO-4 resulted in the formation of RIBO-5. RIBO-5 undergoes cyclization in the presence of TBAF, leading to the production of RIBO-6. The treatment of RIBO-6 with RIBO-7 in the presence of NaCN and MnO_2_ results in the formation of RIBO-8. A Buchwald coupling between RIBO-8 and RIBO-9 ultimately provides RIBO-10. The final step in the synthesis involves the deprotection of RIBO-10 using 6N HCl, resulting in the synthesis of Ribociclib. The synthesis of RIBO-9 begins with the substitution of RIBO-11 with RIBO-12 in *n*-BuOH at 95 °C, yielding RIBO-13. This is followed by treating RIBO-13 with Boc_2_O and K_2_CO_3_ in THF, ultimately affording RIBO-14. The reduction of RIBO-14 provides RIBO-9.

#### 2.8.3. Abemaciclib Mesylate

Abemaciclib Mesylate, branded as Verzenio, received its approval from the FDA in 2017. Abemaciclib is authorized for therapeutic use in the management of advanced or metastatic breast cancer characterized by hormone receptor (HR)-positive, HER2-negative status. It is administered in conjunction with an aromatase inhibitor as an initial endocrine-based treatment for postmenopausal women or men, and in combination with Fulvestrant for women experiencing disease progression following prior endocrine therapy. Through the specific inhibition of CDK4 and CDK6, Abemaciclib disrupts the transition of the cell cycle from the G1 to the S phase, consequently impeding the proliferation of neoplastic cells. In contrast to other CDK inhibitors like Palbociclib and Ribociclib, Abemaciclib demonstrates enhanced specificity toward CDK4 in relation to CDK6 [115]. Within preclinical models, Abemaciclib exhibited antineoplastic efficacy against a spectrum of tumor classifications, encompassing breast cancer cell lines. Its mechanism of action entails the induction of cell cycle arrest and attenuation of tumor progression [116]. The MONARCH 3 trial provided a demonstration of the effectiveness of Abemaciclib. The investigation elucidated a notable enhancement in PFS upon the coadministration of Abemaciclib with an aromatase inhibitor, in contrast to the use of the aromatase inhibitor in isolation, among patients afflicted by advanced breast cancer characterized by HR-positive and HER2-negative status [117]. Common side effects encompass diarrhea, neutropenia, nausea, and fatigue. Serious complications can include venous thromboembolic events and hepatotoxicity.

**Scheme 22:**
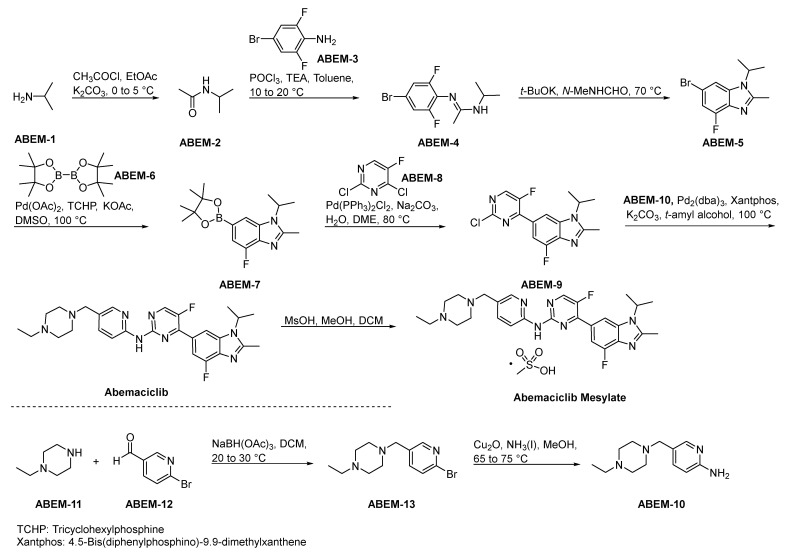
Synthesis of Abemaciclib Mesylate.

The synthesis of Abemaciclib Mesylate commences with the condensation of ABEM-1 with acetyl chloride in the presence of potassium carbonate, yielding the resultant ABEM-2 (Scheme 22). ABEM-2 is subsequently treated with ABEM-3 in the presence of POCl_3_ and TEA, yielding ABEM-4 [118]. The cyclization of ABEM-4 in the presence of *t*-BuOK leads to the formation of ABEM-5. Next, ABEM-7 is prepared through the Pd(II)-catalyzed boronylation of the bromide in the benzimidazole of ABEM-5, utilizing bis(pinacolato)diboron (ABEM-6). The Suzuki coupling of ABEM-7 with ABEM-8 results in the formation of ABEM-9. The synthesis is finalized with the Buchwald–Hartwig coupling of ABEM-9 and ABEM-10, ultimately providing Abemaciclib. The synthesis of ABEM-10 begins with the reductive amination of ABEM-11 with ABEM-12, ultimately yielding ABEM-13. Subsequently, the pyridine bromide of ABEM-13 is replaced through a Pd(0)-catalyzed amination with liquid ammonia and cuprous oxide, resulting in the formation of ABEM-10. Finally, Abemaciclib is treated with MsOH in methanol to yield Abemaciclib Mesylate.

### 2.9. Phosphatidylinositol 3-Kinase (PI3K) Inhibitors

#### Alpelisib

Alpelisib, commercially known as Piqray, received approval from the FDA in 2019. Alpelisib is authorized for utilization in conjunction with Fulvestrant for the therapeutic management of advanced or metastatic breast cancer in postmenopausal women and men. The eligibility criteria for this treatment include the presence of HR-positive, HER2-negative status, along with the identification of PIK3CA mutations through an FDA-approved diagnostic assay. Alpelisib exhibits specificity toward the p110α isoform of PI3K. Its mode of action involves the inhibition of the PI3K pathway, a frequently activated cascade in neoplastic cells, resulting in cellular growth and proliferation. By selectively targeting the p110α isoform, it interrupts this pathway and halts the growth of tumor cells [119,120]. In preclinical models, Alpelisib exhibited robust inhibitory efficacy against cancer cell lines characterized by PIK3CA mutations, alongside its capacity to diminish tumor progression within xenograft models harboring PIK3CA mutations [121]. The SOLAR-1 trial underscored the effectiveness of the Alpelisib and Fulvestrant combination. This regimen elucidated a substantial enhancement in PFS among patients afflicted by advanced breast cancer characterized by PIK3CA, HR-positive, and HER2-negative mutations, in contrast to the administration of Fulvestrant as a monotherapy [122]. Common adverse events include hyperglycemia, rash, nausea, decreased appetite, and fatigue. Serious side effects can encompass severe hypersensitivity and pneumonitis.

**Scheme 23:**
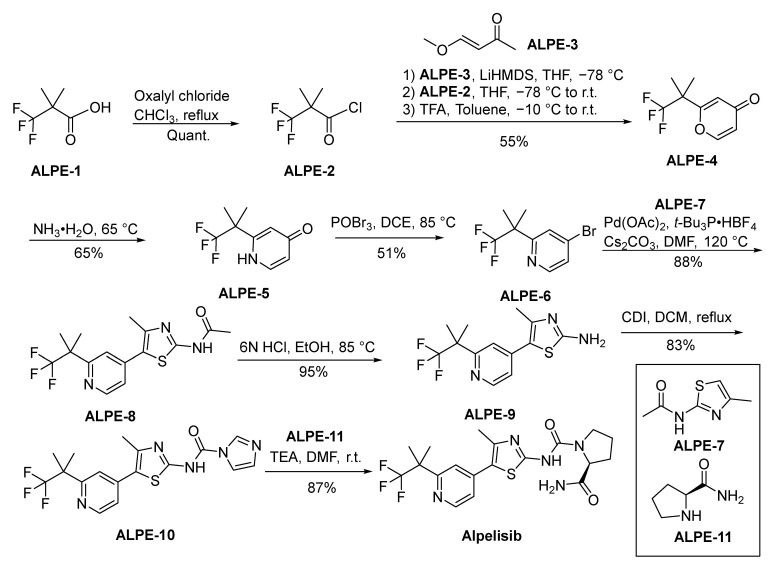
Synthesis of Alpelisib.

The synthesis of Alpelisib commences with the treatment of ALPE-1 with oxalyl chloride in refluxing CHCl_3_, resulting in the formation of ALPE-2 (Scheme 23) [123]. Subsequently, ALPE-3 is treated with lithium bis(trimethylsilyl)amide at −78 °C for a duration of 1 h in THF. Subsequently, ALPE-2 is introduced under the same low-temperature conditions, and the resultant mixture undergoes stirring at ambient temperature for 2.5 h. To this mixture, TFA is incorporated at −10 °C, following which the mixture undergoes further agitation at room temperature, ultimately culminating in the generation of ALPE-4. The treatment of ALPE-4 with aqueous ammonia results in the formation of ALPE-5. ALPE-5 is then subjected to bromination with POBr_3_, affording ALPE-6. The coupling of ALPE-6 with ALPE-7 in the presence of Pd(OAc)_2_, Cs_2_CO_3_ and *t*-Bu_3_P·HBF_4_ in DMF at 120 °C leads to ALPE-8. The deacetylation of ALPE-8 is accomplished by treating it with 6N HCl in ethanol (EtOH) at 80 °C for 1 h, resulting in ALPE-9. Finally, ALPE-9 is treated with N,N-Carbonyldiimidazole (CDI) and ALPE-11 in the presence of TEA, ultimately yielding Alpelisib.

### 2.10. Microtubule Inhibitors

#### 2.10.1. Paclitaxel

Paclitaxel, initially sourced from the Pacific yew tree and commercially available under the trade name Taxol, received regulatory clearance from the FDA in 1992. Paclitaxel is authorized for therapeutic use in patients diagnosed with breast cancer, ovarian cancer, and AIDS-related Kaposi’s sarcoma. Furthermore, it finds application in the management of NSCLC and various other categories of solid tumors. Paclitaxel primarily exerts its influence on intracellular microtubules. It facilitates the formation of microtubules by promoting the assembly of tubulin dimers while concurrently conferring stability upon these microtubules through the inhibition of depolymerization. This augmented stability disrupts the customary dynamic reorganization of the microtubular network, which plays a pivotal role in essential interphase and mitotic cellular processes. In essence, it induces cell cycle arrest at the G2/M phase, thereby impeding cellular replication [124]. In preclinical models, Paclitaxel exhibited antitumor properties against several tumor types, halting cell division and inducing apoptosis [125]. Several clinical trials have showcased Paclitaxel’s efficacy in breast, ovarian, and NSCLC, leading to improved response rates and, in some cases, enhanced survival [126]. Adverse effects include neutropenia, neuropathy, joint and muscle pain, cardiotoxicity, and hypersensitivity reactions. It is crucial to pre-medicate to prevent severe hypersensitivity [127].

**Scheme 24:**
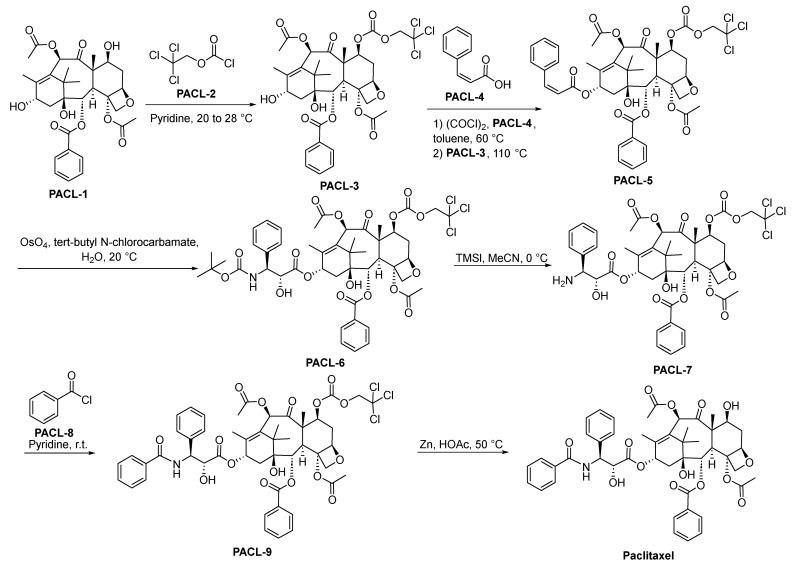
Synthesis of Paclitaxel.

The synthesis of Paclitaxel commences with the regioselective esterification of PACL-1 using PACL-2 in pyridine, resulting in the formation of PACL-3 (Scheme 24). Subsequently, PACL-3 undergoes esterification with PACL-4 in the presence of oxalyl chloride, leading to the generation of PACL-5 [128]. Next, the oxidative addition of tert-butyl N-chlorocarbamate to PACL-5 by means of OsO_4_ in water affords the protected amino hydroxyester PACL-6, which is further subjected to deprotection of the Boc group using TMSI, ultimately affording PACL-7. The condensation of PACL-7 with PACL-8 in pyridine gives rise to PACL-9. The final step in the synthesis involves reductive deprotection of PACL-9 using zinc powder in acetic acid, resulting in the synthesis of Paclitaxel.

#### 2.10.2. Vinorelbine Ditartrate

Vinorelbine Ditartrate, marketed under the trade name Navelbine, received regulatory clearance from the FDA in 1994. Vinorelbine finds its principal clinical application in the initial treatment of individuals diagnosed with metastatic NSCLC, in addition to its utilization in cases of advanced or metastatic breast cancer subsequent to the ineffectiveness of prior chemotherapeutic interventions. Like other vinca alkaloids, Vinorelbine targets the microtubules in cells [129]. Vinorelbine exerts its mechanism of action through the binding to tubulin, thereby impeding the formation of microtubules and thwarting the elongation of these structures. This interference results in the perturbation of the mitotic spindle, thereby inducing cell cycle arrest at the metaphase stage and subsequently precipitating apoptosis. Preclinical studies demonstrated that Vinorelbine possesses antitumor activity against an extensive spectrum of tumor models, inhibiting cell proliferation and inducing cell death [130]. Clinical trials have shown the efficacy of Vinorelbine in treating NSCLC, with improved overall survival and response rates in comparison to other treatments. It has also been deemed effective as part of combination therapies for breast cancer [131]. The prevailing adverse effects frequently encompass neutropenia, fatigue, constipation, and nausea. Additionally, the manifestation of neurotoxicity, which is typified by peripheral neuropathy, may also transpire [132].

**Scheme 25:**
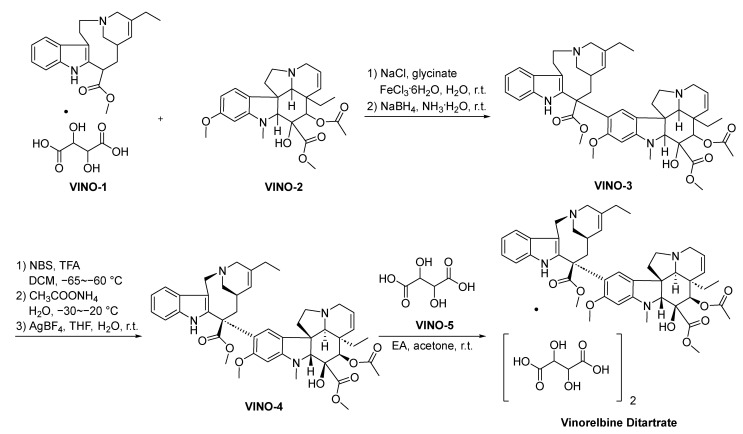
Synthesis of Vinorelbine Ditartrate.

The synthesis of Vinorelbine Ditartrate is initiated by treating VINO-1 and VINO-2 with NaCl, glycinate, and FeCl_3_·H_2_O (Scheme 25). This is followed by reduction in NaBH_4_, resulting in the formation of VINO-3 [133]. Subsequently, VINO-3 undergoes bromination with NBS in the presence of trifluoroacetic acid (CF_3_COOH) under cold conditions, followed by a rearrangement step in the presence of ammonium acetate, AgBF_4_, and NaHCO_3_, leading to vinorelbine crude product VINO-4. Finally, VINO-4 undergoes repeated column purification processes, followed by treatment with tartaric acid (VINO-5) and recrystallization, resulting in the isolation of Vinorelbine Ditartrate.

#### 2.10.3. Docetaxel

Docetaxel, commercially available under the trade name Taxotere, attained regulatory clearance from the FDA on 14 May 1996. Docetaxel exhibits clinical indications across a spectrum of malignancies, encompassing breast cancer, prostate cancer, NSCLC and head and neck cancers. Docetaxel primarily targets the microtubules within cells. Docetaxel achieves microtubule stabilization through its interaction with the β-tubulin subunit, thus impeding the process of depolymerization. This event culminates in cell cycle arrest, specifically at the G2/M phase, ultimately precipitating apoptotic cell death [134]. Preclinical investigations have unveiled the robust antineoplastic potential of Docetaxel within a diverse array of tumor models, including those that have demonstrated resistance to paclitaxel, another member of the taxane class [135]. Clinical trials have provided empirical validation of Docetaxel’s therapeutic efficacy, notably in the context of metastatic breast cancer and NSCLC. Whether employed within combination regimens or as a standalone intervention, it has demonstrated enhancements in both survival metrics and response rates [136]. Common side effects encompass neutropenia, fatigue, fluid retention, neuropathy, and alopecia. Severe hypersensitivity reactions may also occur.

**Scheme 26:**
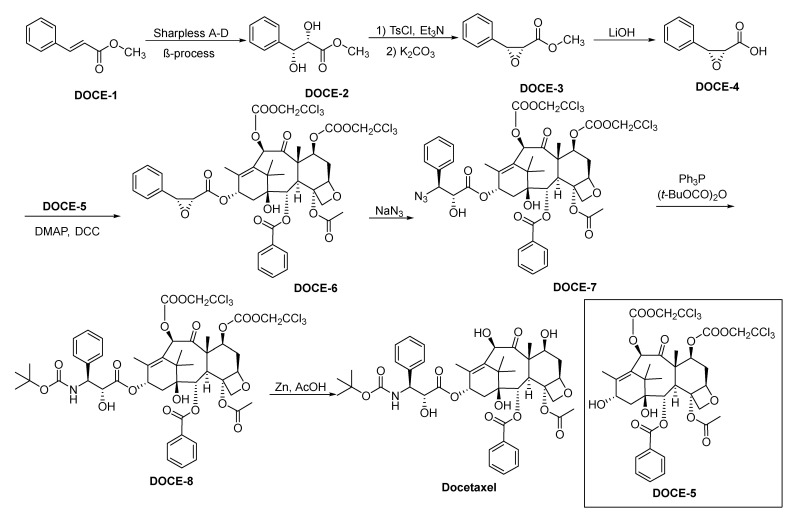
Synthesis of Docetaxel.

The synthetic route of Docetaxel is initiated through the application of the Sharpless AD process (AD-B: The process typically involves the use of osmium tetroxide as the oxidizing agent along with a chiral ligand, such as the popular ligand (DHQD)_2_PHAL, to induce chirality in the dihydroxylation reaction) to DOCE-1, thereby effecting the formation of DOCE-2 (Scheme 26) [137]. Subsequently, the diol DOCE-2 undergoes a selective conversion into the α-tosylate derivative. This compound, upon exposure to K_2_CO_3_ in an aqueous DMF environment, results in the generation of DOCE-3 with a moderate yield. Subsequent hydrolysis of DOCE-3 with LiOH produces DOCE-4. DOCE-4 undergoes esterification with DOCE-5 under the influence of DCC and DMAP, conducted within a toluene milieu at 80 °C, culminating in the production of DOCE-6 with an impressive yield of 91%. DOCE-6, characterized by its epoxide functionality, undergoes a chemical transformation by reacting with NaN_3_ in an aqueous MeOH solution, facilitated by the presence of methyl formate, at an elevated temperature of 50 °C, ultimately yielding the azide derivative DOCE-7. DOCE-7, having been previously synthesized, undergoes a subsequent chemical transformation. It was subjected to reaction with PPh_3_, within the milieu of CH_2_Cl_2_, in the presence of Boc_2_O, K_2_CO_3_, and a minor proportion of water. This intricate process culminates in the successful generation of DOCE-8. The final step in the synthesis involves reductive deprotection of DOCE-8 using zinc powder in a mixture of acetic acid and MeOH, yielding Docetaxel.

#### 2.10.4. Ixabepilone

Ixabepilone, marketed under the brand name Ixempra, received regulatory approval from the FDA in 2007. Its approved indications encompass the treatment of metastatic or locally advanced breast cancer, whether employed as a standalone therapeutic agent or in combination with Capecitabine. This treatment regimen is specifically intended for patients who have experienced disease progression following the administration of anthracyclines, taxanes, and Capecitabine [138]. Ixabepilone specifically targets microtubules in cancer cells. Functioning similarly to taxanes, Ixabepilone binds to β-tubulin on microtubules, stabilizing and preventing their disassembly. This perturbs the conventional cellular mitotic progression, culminating in the sequestration of cells within the G2/M phase and the instigation of apoptotic cascades [139,140]. In preclinical investigations, Ixabepilone exhibited robust antineoplastic efficacy across a spectrum of malignancies, encompassing multidrug-resistant cell lines and taxane-resistant counterparts [141]. Clinical studies have provided empirical evidence of the effectiveness of Ixabepilone, particularly in the context of metastatic breast carcinoma. Its advantages encompass enhanced response rates and protracted progression-free survival, particularly when employed concomitantly with Capecitabine [142]. Common side effects encompass neuropathy, fatigue, myalgia, and alopecia. Neutropenia is a significant hematological adverse effect.

**Scheme 27:**
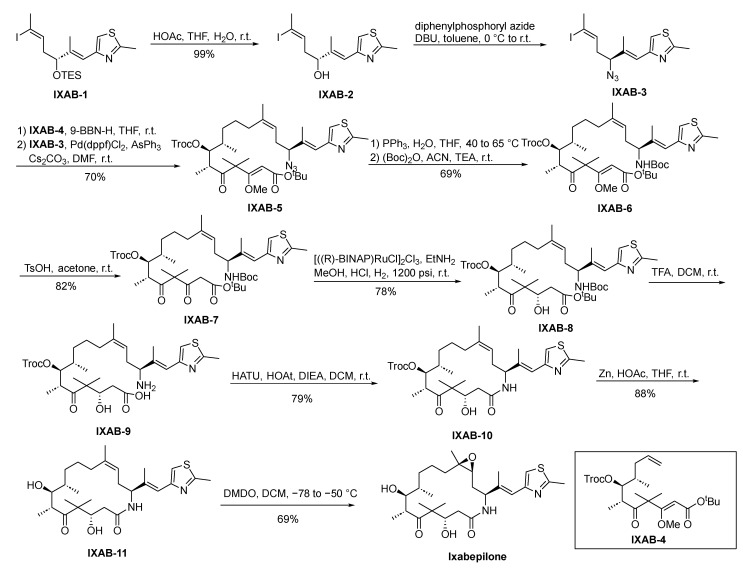
Synthesis of Ixabepilone.

The synthesis of Ixabepilone commences with the deprotection of the silyl ether group in IXAB-1 using a mixture of HOAc, THF, and water, resulting in the formation of IXAB-2 (Scheme 27) [143]. The free alcohol IXAB-2 is then transformed into the corresponding azide, IXAB-3, utilizing 1,8-diazabicyclo[5.4.0]undec-7-ene (DBU) and diphenylphosphoryl azide. After this, the Suzuki coupling of IXAB-3 with IXAB-4 gives rise to IXAB-5. The next step involves Staudinger reduction of IXAB-5, followed by treatment with Boc_2_O and TEA, ultimately affording IXAB-6. IXAB-6 is further treated with *p*-TsOH in acetone, yielding IXAB-7. IXAB-7 undergoes a ruthenium-mediated asymmetric hydrogenation process in methanol employing a modified Noyori catalyst, yielding IXAB-8. Concomitant removal of the tert-butyl carbamate and tert-butyl ester moieties is successfully accomplished through the application of TFA in a dichloromethane solvent system, resulting in the synthesis of amino acid derivative IXAB-9. The intramolecular condensation of IXAB-9 gives rise to IXAB-10. Subsequently, deprotection of the Troc group in IXAB-10 is accomplished by subjecting it to sonication with zinc dust, yielding IXAB-11. Finally, IXAB-11 is epoxidized using 2,2-dimethyldioxirane at −50 °C, resulting in the formation of Ixabepilone.

#### 2.10.5. Eribulin

Eribulin, commercially recognized as Halaven, garnered approval from the FDA in 2010. Eribulin finds clinical indication in the management of metastatic breast cancer among patients who have undergone a minimum of two prior chemotherapeutic protocols for metastatic disease, inclusive of both anthracycline and taxane regimens [144]. It is also approved for certain types of liposarcoma. Eribulin targets microtubules, essential components of the cytoskeleton, and mitotic spindle in cells. Eribulin exerts its pharmacological action by selectively restraining microtubule elongation while leaving microtubule shortening unaffected. This functional attribute culminates in cell cycle arrest at the G2/M phase, consequently facilitating the initiation of programmed apoptotic cell demise. This action is distinct from other tubulin-targeting agents [145]. Preclinical investigations have substantiated Eribulin’s pronounced antineoplastic potential across a diverse spectrum of cancer cell lines, encompassing those that exhibit resistance to alternative therapeutic agents. Its unique mechanism of action gave it an edge over other microtubule-targeting drugs [146]. In clinical trials, Eribulin exhibited a statistically notable enhancement in overall survival among patients afflicted with metastatic breast cancer, surpassing the outcomes achieved with alternative therapeutic modalities [147]. Notable side effects encompass neutropenia, fatigue, alopecia, peripheral neuropathy, and nausea. Rare, but severe side effects like QT prolongation can also occur.

**Scheme 28:**
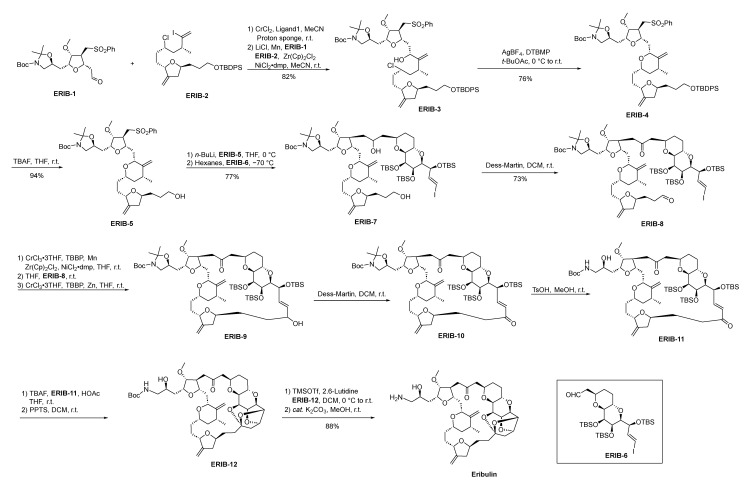
Synthesis of Eribulin.

The synthesis of Eribulin is initiated with the Nozaki–Hiyama–Kishi reaction of ERIB-1 with ERIB-2 catalyzed by CrCl_2_, LiCl, Zr(Cp)_2_Cl_2_, and a complex of nickel(II) chloride with 2,9-dimethyl-1,10-phenanthroline (NiCl_2_-dmp) in acetonitrile, yielding ERIB-3. This is followed by treatment with AgBF_4_, 2,6-Di-tert-butyl-4-methylpyridine (DTBMP), and *t*-BuOAc, ultimately providing ERIB-4 (Scheme 28) [148]. Subsequently, ERIB-4 is subjected to treatment with tetra-butyl ammonium fluoride (TBAF) in THF, resulting in ERIB-5. This compound, ERIB-5, is further treated with *n*-BuLi and ERIB-6 to yield ERIB-7. The Dess–Martin oxidation of ERIB-7 leads to ERIB-8, which is further subjected to intramolecular Nozaki–Hiyama–Kishi reaction to generate ERIB-9. A subsequent Dess–Martin oxidation of ERIB-9 yields ERIB-10, which, upon treatment with *p*-TsOH in MeOH, provides ERIB-11. The intramolecular cyclization of ERIB-11 is carried out in the presence of TBAF, HOAc, and pyridinium PPTS, yielding ERIB-12. The final step in the synthesis involves the treatment of ERIB-12 with TMSOTf, 2,6-lutidine, and K_2_CO_3_, ultimately yielding Eribulin.

### 2.11. Poly (ADP-Ribose) Polymerase (PARP) Inhibitor

#### Talazoparib

Talazoparib, marketed as Talzenna, received approval from the FDA in 2018. Talazoparib is approved for the therapy of adult individuals diagnosed with locally advanced or metastatic breast cancer that is HER2-negative and is associated with deleterious or suspected deleterious germline mutations in the breast cancer susceptibility gene (BRCA) [149]. Talazoparib serves as a highly effective inhibitor of PARP enzymes. Its mechanism of action involves the inhibition of PARP enzymes, leading to the impairment of the repair process for single-strand DNA breaks. Notably, in cells bearing BRCA mutations, Talazoparib exerts a dual impact by inhibiting PARP and exploiting the non-functionality of BRCA. This synergy culminates in the accumulation of DNA damage, ultimately driving cellular apoptosis [150]. In preclinical models, Talazoparib demonstrated significant antitumor activity, especially in BRCA1- and BRCA2-deficient tumors, with its dual mechanism of trapping PARP-DNA complexes and preventing DNA repair [151,152]. In the context of the EMBRACA phase III trial, Talazoparib demonstrated a noteworthy extension in progression-free survival among patients afflicted with advanced breast cancer harboring BRCA1/2 mutations when juxtaposed with conventional chemotherapy regimens [153]. Common side effects encompass fatigue, anemia, neutropenia, and nausea. More serious risks include the potential for myelodysplastic syndrome or acute myeloid leukemia.

**Scheme 29:**
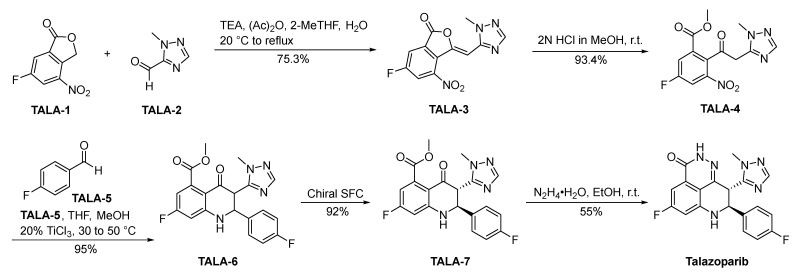
Synthesis of Talazoparib.

The synthesis of Talazoparib commences with the Knoevenagel condensation of TALA-1 and TALA-2, resulting in the formation of TALA-3 (Scheme 29). This is followed by treatment with 2N HCl in MeOH, yielding TALA-4 [154]. Subsequently, TALA-4 undergoes cyclization with TALA-5 in the presence of TiCl_3_, giving rise to TALA-6. Chiral resolution of TALA-6 leads to the isolation of TALA-7. The chiral resolution of TALA-6 is carried out on a SFC unit with a CHIRALPAKRIC 3 cm (I.D.) × 25 cm, 5 µm column, using CO_2_/MeOH (80/20) as a mobile phase at a flow rate of 65 g/min while maintaining the column temperature at 35 °C, and with a detection UV wavelength of 254 nm. The final step involves the cyclization of TALA-7 in the presence of hydrazine hydrate (N_2_H_4_·H_2_O) in EtOH, ultimately resulting in the synthesis of Talazoparib.

## 3. Conclusions

The future prospects of small-molecule development are promising, with advancements in computational techniques and high-throughput screening methods enabling more efficient drug discovery. Small-molecules continue to offer advantages such as oral bioavailability and target specificity, making them valuable candidates for treating a wide range of diseases. Additionally, innovations in synthetic chemistry and molecular design are enhancing the potency and selectivity of small-molecule drugs, driving further progress in pharmaceutical research and development. In the ever-evolving landscape of breast cancer therapeutics, small-molecule drugs have emerged as a beacon of innovation, offering targeted solutions to a historically challenging disease. Through meticulous scrutiny of their synthesis, mechanisms of action, and clinical utility, several salient points become evident. Firstly, the synthetic pathways employed to produce these compounds are a testament to the strides made in modern medicinal chemistry. Leveraging advanced methodologies, often with a focus on chiral selectivity and optimization of yield, these pathways underscore the confluence of creativity and precision. Furthermore, the consistent evolution of these synthetic approaches not only ensures the production of more effective molecules but also hints at a future where customized synthesis tailored to specific patient profiles may become feasible. Mechanistically, small-molecules have illuminated our understanding of the intricate molecular intricacies of breast cancer. By selectively targeting kinases, modulating hormone receptor functions, or thwarting DNA repair mechanisms, they offer targeted disruption of oncogenic pathways. This specificity, in addition to ensuring therapeutic efficacy, provides invaluable insights into the biology of breast cancer, guiding future drug discovery endeavors. From a clinical vantage point, the tangible benefits of these drugs are undeniable. Improved survival rates, reduced recurrence, and, notably, the potential to overcome drug-resistant phenotypes, have reshaped the treatment narrative.

However, the journey is not devoid of challenges. As with all therapeutic modalities, resistance mechanisms, unforeseen side effects, and patient stratification remain areas demanding attention. New targets and pathways have emerged as promising avenues for understanding and combating breast cancer. Bromodomain-containing protein 4 (BRD4) regulates gene transcription and has been implicated in breast cancer progression by promoting cell proliferation and survival [155]. Polo-like kinase 1 (PLK1) plays a crucial role in cell cycle regulation and its overexpression correlates with poor prognosis in breast cancer patients [156]. Programmed death-ligand 1 (PD-L1) expression on tumor cells enables immune evasion and is being targeted with immunotherapy in breast cancer treatment [157]. Histone deacetylases (HDACs) alter chromatin structure and gene expression, contributing to breast cancer development [158]. The PI3K/AKT/mTOR pathway regulates cell growth and survival, frequently dysregulated in breast cancer, making it an attractive therapeutic target [159]. In summation, while the advent of small-molecule drugs for breast cancer treatment is undoubtedly transformative, it is just a chapter in the ongoing saga of breast cancer research. It is incumbent upon the scientific community to harness the learnings from these molecules, address extant challenges, and pave the way for a future where breast cancer, in all its forms, can be effectively managed, if not entirely eradicated.

## Figures and Tables

**Table 1:** Small-molecule drugs for the treatment of breast cancer.

NO	Drug Name	Original Company	Target	Brand Name	Approval Year
1	Methotrexate	Lederle Laboratories	DNA	Emtexate	1953
2	Megestrol Acetate	Bristol-Myers Squibb	Progesterone receptor	Megace	1971
3	Doxorubicin Hydrochloride	Pfizer	DNA	Adriamycin	1974
4	Tamoxifen Citrate	AstraZeneca	Estrogen receptor	Nolvadex	1977
5	Aminoglutethimide	Novartis	Aromatase enzyme	Cytadren	1980
6	Paclitaxel	Bristol-Myers Squibb	Microtubule	Taxol	1992
7	Vinorelbine Ditartrate	GlaxoSmithKline	Microtubule	Navelbine	1994
8	Anastrozole	AstraZeneca	Aromatase enzyme	Arimidex	1995
9	Docetaxel	Sanofi-Aventis	Microtubule	Taxotere	1996
10	Gemcitabine	Eli Lilly	DNA	Gemzar	1996
11	Letrozole	Novartis	Aromatase enzyme	Femara	1997
12	Toremifene	Orion Corporation	Estrogen receptor	Fareston	1997
13	Raloxifene	Eli Lilly	Estrogen receptor	Evista	1997
14	Capecitabine	Roche	DNA	Xeloda	1998
15	Epirubicin Hydrochloride	Pfizer	DNA	Ellence	1999
16	Exemestane	Pfizer	Aromatase enzyme	Aromasin	1999
17	Fulvestrant	AstraZeneca	Estrogen receptor	Faslodex	2002
18	Lapatinib Ditosylate	GlaxoSmithKline	HER2 receptor tyrosine kinase	Tykerb	2007
19	Ixabepilone	Bristol-Myers Squibb	Microtubule	Ixempra	2007
20	Eribulin	Eisai	Microtubule	Halaven	2010
21	Palbociclib Hydrochloride	Pfizer	Cyclin-dependent kinases 4 and 6 (CDK4/6)	Ibrance	2015
22	Ribociclib	Novartis	Cyclin-dependent kinases 4 and 6 (CDK4/6)	Kisqali	2017
23	Abemaciclib Mesylate	Eli Lilly	Cyclin-dependent kinases 4 and 6 (CDK4/6)	Verzenio	2017
24	Neratinib	Puma Biotechnology	HER2 receptor tyrosine kinase	Nerlynx	2017
25	Talazoparib	Pfizer	Poly (ADP-ribose) polymerase (PARP)	Talzenna	2018
26	Alpelisib	Novartis	Phosphatidylinositol 3-kinase (PI3K)	Piqray	2019
27	Tucatinib	Seattle Genetics	HER2 receptor tyrosine kinase	Tukysa	2020
28	Fluoroestradiol F-18	PETNET Solutions Inc.	Estrogen receptor	Detectnet	2020
29	Elacestrant	Radius Health	Estrogen receptor	Orserdu	2023

## Data Availability

Data are contained within the article.
